# The BR domain of PsrP interacts with extracellular DNA to promote bacterial aggregation; structural insights into pneumococcal biofilm formation

**DOI:** 10.1038/srep32371

**Published:** 2016-09-01

**Authors:** Tim Schulte, Cecilia Mikaelsson, Audrey Beaussart, Alexey Kikhney, Maya Deshmukh, Sebastian Wolniak, Anuj Pathak, Christine Ebel, Jonas Löfling, Federico Fogolari, Birgitta Henriques-Normark, Yves F. Dufrêne, Dmitri Svergun, Per-Åke Nygren, Adnane Achour

**Affiliations:** 1 Science for Life Laboratory, Department of Medicine Solna, Karolinska Institute, and Department of Infectious Diseases, Karolinska University Hospital, Solna, SE-17176 Stockholm, Sweden; 2 Université catholique de Louvain, Institute of Life Sciences, Croix du Sud, 4-5, bte L7.07.06, B-1348 Louvain-la-Neuve, Belgium; 3 European Molecular Biology Laboratory (EMBL), Hamburg Outstation, Notkestrasse 85, 22603 Hamburg, Germany; 4 Department of Microbiology, Tumor and Cell Biology (MTC), Karolinska Institute; Clinical Microbiology, Karolinska University Hospital Solna, Stockholm, Sweden; 5 Institut de Biologie Structurale (IBS), Univ. Grenoble Alpes, CEA, CNRS, 38044 Grenoble, France; 6 Dipartimento di Scienze Mediche e Biologiche, Universita’ di Udine, Piazzale Kolbe 4, 33100 Udine - Italy; 7 Division of Protein Technology, School of Biotechnology, KTH–Royal, Institute of Technology, Sweden

## Abstract

The major human pathogen *Streptococcus pneumoniae* is a leading cause of disease and death worldwide. Pneumococcal biofilm formation within the nasopharynx leads to long-term colonization and persistence within the host. We have previously demonstrated that the capsular surface-associated pneumococcal serine rich repeat protein (PsrP), key factor for biofilm formation, binds to keratin-10 (KRT10) through its microbial surface component recognizing adhesive matrix molecule (MSCRAMM)-related globular binding region domain (BR_187–385_). Here, we show that BR_187–385_ also binds to DNA, as demonstrated by electrophoretic mobility shift assays and size exclusion chromatography. Further, heterologous expression of BR_187–378_ or the longer BR_120–378_ construct on the surface of a Gram-positive model host bacterium resulted in the formation of cellular aggregates that was significantly enhanced in the presence of DNA. Crystal structure analyses revealed the formation of BR_187–385_ homo-dimers via an intermolecular β-sheet, resulting in a positively charged concave surface, shaped to accommodate the acidic helical DNA structure. Furthermore, small angle X-ray scattering and circular dichroism studies indicate that the aggregate-enhancing N-terminal region of BR_120–166_ adopts an extended, non-globular structure. Altogether, our results suggest that PsrP adheres to extracellular DNA in the biofilm matrix and thus promotes pneumococcal biofilm formation.

The human adapted, Gram-positive commensal bacterium *Streptococcus pneumoniae* (pneumococcus) colonizes the upper respiratory tract in about 10% of healthy adults and up to 60% of children without necessarily causing any symptoms^1^. The organization of bacterial communities into complex and dynamic biofilm structures allows for growth in a hostile environment, providing protection against anti-microbial agents^2^,^3^. Pneumococcal colonization is the precursor for otitis media and sinusitis, as well as severe diseases including pneumonia, septicemia and meningitis^4^. Pneumococcal biofilms are found on the surface of adenoid and mucosal epithelial tissues in children with recurrent middle-ear infections and otitis media with effusion, highlighting the role of microbial biofilms in disease development^5^.

Recently, studies using *in vitro* and *in vivo* pneumococcal biofilm models have begun to unveil the biogenesis, regulation, structure and composition of pneumococcal biofilms^6^,^7^,^8^. The exact composition of the pneumococcal biofilm extracellular polymer matrix (EPM) depends largely on the species and environmental conditions^5^,^9^. Most often, the EPM is composed of exo-polysaccharides, proteins, nucleic acids and lipids, and its overall structure is stabilized through intermolecular interaction networks, often involving non-catalytic carbohydrate- or eDNA-binding proteins such as lectins and pilins^8^,^10^. In pneumococcus, the amount of capsular polysaccharides is highly regulated and significantly reduced during colonization compared to invasion^1^. Non-encapsulated strains are hyper-adhesive to epithelial cells and form biofilms more efficiently than encapsulated strains. On the other hand, they are also less virulent^5^,^11^,^12^,^13^. The increased adhesive phenotype in non-encapsulated strains can possibly be attributed to an increased presentation of adhesion proteins^13^,^14^.

The pneumococcal serine rich repeat protein (PsrP), present in 60% of strains capable of causing pneumonia in children, has been identified as a key factor for biofilm formation using *in vitro* and *in vivo* models^7^,^15^. A pneumococcal ΔPsrP deletion mutant strain was unable to form biofilm aggregates in an *in vivo* mice colonization model and elicited a significantly enhanced immune response compared to the wild-type strain^7^. PsrP belongs to the serine rich repeat protein (SRRP) family found in Gram-positive bacteria. SRRPs are attached to the capsular surface via a cell wall anchoring domain and display a long, highly repetitive and glycosylated C-terminal serine rich-repeat (SRR) region. The SRR region varies between 400 and 4000 residues in length, extending the functional binding region (BR) domain out of the capsule^15^,^16^,^17^,^18^ (Fig. 1A). SRRP BR domains which bind to a broad range of targets including glyco-conjugates and keratins, are variable in sequence and organized into modular domains^17^,^18^. Fold topologies similar to the microbial surface component recognizing adhesive molecule (MSCRAMM) CnaA from *Staphylococcus aureus* were determined for domains of the SRRPs GspB and Fap1 from *Streptococcus parasanguinis* and *Streptococcus gordonii*, respectively, and also predicted for domains of four other SRRPs^16^,^17^,^19^. Although the fold topology of the BR domain of PsrP compared to the other BR domains could not be predicted due to very low sequence similarity^17^, the recently determined crystal structure of the KRT10-binding domain of PsrP (BR_187–385_) revealed a topology distantly related to the CnaA fold^20^. Interestingly, GspB, Fap1 and PsrP, among several other SRRPs, were demonstrated to mediate intra-species interactions during biofilm formation^15^,^17^,^18^,^21^,^22^. A molecular model for the oligomerization of the helical subdomain NRα of Fap1 has also been proposed based on higher oligomers found in the crystal lattice and predicted as stable in solution^21^. The suggested molecular model is one of only a few known examples of biofilm-associated protein oligomerizations for which high-resolution structural data exist^23^. Recently, the crystal structures of the *Staphylococcus aureus* surface protein G (SasG) and of the accumulation-associated protein (Aap) from *Staphylococcus epidermidis*, both essential in biofilm formation, have been determined^24^,^25^,^26^,^27^. Like PsrP, these homologous multi-domain proteins are covalently attached to the peptidoglycan via their LPxTG motifs. Dimerization of the Aap B-repeat domains requires upstream proteolytic cleavage and a similar mechanism has also been suggested for SasG^24^,^26^. Crystal structures of self-oligomerizing B-repeat regions of both proteins revealed remarkable “free-standing” β-sheet topologies^25^,^27^. Based on the Aap B-domain crystal structure, a model for the Zn^2+^-dependent dimerization of the entire B-repeat domains was suggested according to which two molecules are wrapped around each other like entangled fibers in a rope^27^. Although a molecular understanding of the Zn^2+^-dependent dimerization of the MSCRAMM Fibronectin-Binding Protein A (FnBPA) of *S. aureus* is still lacking, atomic force microscopy (AFM) experiments elegantly demonstrated that cellular aggregation can be induced through multiple low-affinity homophilic bonds between copies of a single protein^28^.

The self-oligomerization property of PsrP has previously been suggested to occur through residues 122–166 (BR_122–166_), preceding the KRT10-binding region (BR_187–385_)^15^. However, a molecular mechanism underlying PsrP oligomerization has so far not been provided. In this study, crystal structure analysis and atomic force microscopy experiments revealed homophilic low affinity interactions, through the formation of an intermolecular β-sheet between two BR_187–385_ molecules. Electrophoretic mobility shift assays demonstrated that BR_187–385_ binds efficiently to DNA. Importantly, heterologous display of BR_187–385_ on the surface of *Staphylococcus carnosus* demonstrated the capacity of BR_187–385_ to promote eDNA-dependent cellular aggregation. Small-angle X-ray scattering and circular dichroism experiments revealed a disordered and non-globular structure in the region corresponding to BR_122–166_. We here demonstrate that BR_122–166_ is released from PsrP through proteolytic cleavage by the human furin protease, a known maturation factor of other toxins and virulence factors. The discovered molecular mechanisms provide novel structural and mechanistic insights into the role of PsrP during pneumococcal biofilm formation.

## Results and Discussion

### The BR domain comprises a furin protease recognition site and forms irreversibly associated dimers only during heterologous expression

**Figure 1:**
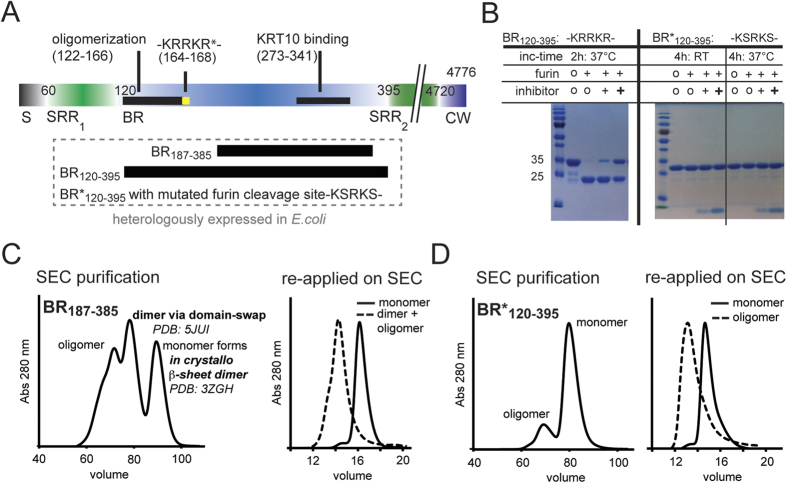
The BR domain comprises a furin protease recognition site and forms irreversibly associated dimers only during heterologous expression. (**A**) PsrP is organized into five domains: the N-terminal signal sequence (S) for extracellular translocation of PsrP, two putatively glycosylated serine rich repeat regions (SRR_1_ and SRR_2_), the binding region (BR) domain and the cell wall domain (CW). BR harbors two distinct sub-regions for KRT10 binding (black bar, residues 273–341) and self-oligomerization (black bar, residues 122–166), respectively. The sequence K_164_RRKR_168_ is recognized by the human Furin protease. The three constructs BR_120–395_, BR_187–385_ and BR_187–378_ used within this study are displayed below the overall schematic representation of PsrP. In the mutated version of BR_120–395_ (BR*_120–395_), KRRKR was substituted to KSRKS. (**B**) A protease cleavage assay confirmed that furin recognized the KRRKR motif, but did not cleave in the presence of the furin protease inhibitor or when the KRRKR sequence was substituted to KSRKS. Cleavage was performed at room temperature (RT) and 37 °C for the indicated incubation times. The inhibitor was used at 100 μM and 250 μM, shown as plus signs in regular and bold format, respectively. (**C**) Distinct populations of BR_187–385_ were eluted using Superdex 200 HiLoad 16/600 at elution column volumes (CV) of 89, 78, 71 and 66 mL corresponding to apparent molecular weights of 22, 55 and 90, 150 kDa, as well as higher oligomers. In this study, we show that the irreversibly associated dimer of BR_187–385_ is formed through a domain swap mechanism (PDB: 5JUI, Fig. S2) and that a low-affinity β-sheet dimer is created between two symmetry-related molecules in the previously determined crystal structure of the BR_187–385_ monomer (PDB: 3ZGH, Fig. 3). (**D**) BR*_120–395_ was eluted from the same column at CV of 79 and 69 mL corresponding to apparent MW of 50 and 115 kDa. (**C**,**D**) When the isolated monomer and oligomer populations of BR_187–385_ and BR*_120–395_ were re-applied on analytical Superdex 200 HR10/30 columns, the monomer and oligomer populations were stable and did not interconvert between each other.

Sequence analysis and biochemical protease cleavage assays revealed that the sequence motif K_164_RRKR_168_, localized between the KRT10-binding region^20^ and the predicted non-globular N-terminal extension BR_120–166_, is cleaved by the human furin protease (Fig. 1A,B). While wild-type BR_120–395_ was specifically cleaved by the human furin protease and prone to degradation during purification, a BR*_120–395_ construct with a mutated furin site was not cleaved and significantly more stable compared to wild-type, with no degradation product observed during purification (data not shown). Furin-like proteases activate a large number of pro-protein substrates as well as bacterial and viral pathogenic agents^29^,^30^. Thus, we hypothesized that the N-terminal fragment comprising BR_120–166_ and SRR_1_ could be released following proteolysis by furin, similarly to the previously described shedding of the glycoprotein Flo11p from *Saccharomyces cerevisiae* cells into the extracellular matrix of yeast mats^31^.

We have recently determined the crystal structure of the KRT10-binding domain of PsrP (BR_187–385_), revealing a novel MSCRAMM-related DEv-IgG fold resembling a compressed barrel^20^. The crystals were obtained from a monomeric preparation of BR_187–385_ that did not form higher oligomers in solution as assessed by analytical ultracentrifugation (AUC) and small angle X-ray scattering (SAXS) experiments at different concentrations^20^. However, during purification of the heterologously produced BR_187–385_, higher oligomer states were formed in the final size exclusion chromatography (SEC) step (Fig. 1C). Interestingly, these isolated oligomer populations were stable in solution but not in steady-state equilibrium with the monomer, as demonstrated using AUC and SEC (Figs 1C and S1A, Table S1). Similarly, the larger BR_120–395_ construct produced stable higher oligomer species, but at a significantly reduced ratio (Figs 1D and S1B, Table S1).

Since stable oligomer formation of PsrP could be relevant for pneumococcal biofilm formation, crystallization trials were set up for the isolated BR_187–385_ dimer in order to obtain a molecular understanding for stable dimer formation. However, crystals were hard to reproduce and those obtained diffracted poorly. Therefore, a shorter construct was designed by deleting the seven C-terminal residues in BR_187–385_ that were not built in the previously determined crystal structure due to missing electron density^20^. Well-diffracting crystals were obtained of the isolated irreversibly associated BR_187–378_ dimer. Analysis of the crystal structure solved by molecular replacement revealed that the residue stretches L202-G315 (region I) and Y316-S377 (region II) are reciprocally exchanged between two separate chains (Fig. S2, Table S2). This reciprocal exchange of entire protein segments is termed 3D-domain swapping and has been first described for diphtheria toxin^32^,^33^. A common feature of domain-swapped oligomers is the requirement to pass a high-energy barrier between the non-swapped and swapped states. Such inter-conversions usually do not occur spontaneously, but require harsh and denaturing conditions^32^. Thus, it should be noted that the irreversibly associated dimers were only obtained following heterologous production of the isolated BR protein constructs, and that, at this stage, we remain uncertain about their formation *in vivo*.

### The putative self-oligomerization region BR_122–166_ is non-globular and does not promote self-aggregation

**Figure 2:**
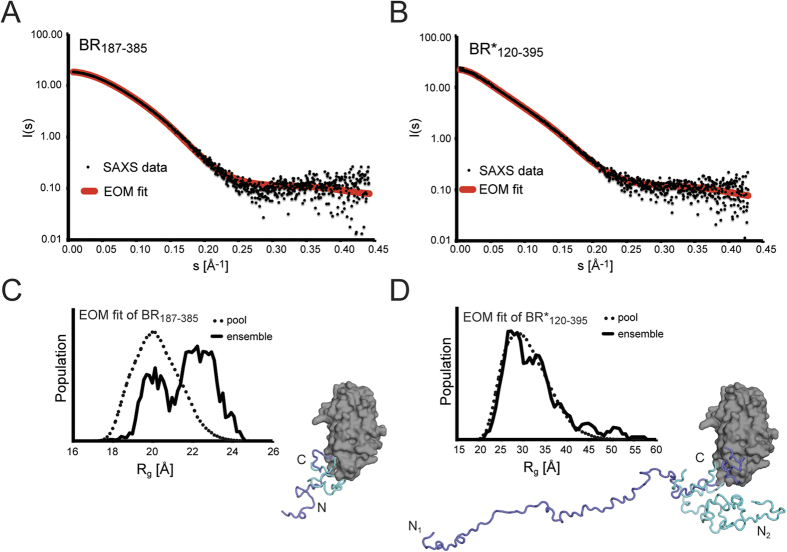
The N-terminal region BR_120–186_ forms a non-globular, flexible structure as revealed by SAXS. (**A**,**B**) Processed solution scattering data (black dots) from (**A**) BR_187–385_ and (**B**) BR*_120–395_ fit to the theoretical scattering curves (red lines) from ensembles of 50 monomer models obtained from the ensemble optimization method (EOM) with χ-values of 0.9 and 1.7. (**C**,**D**) The histograms of the radii of gyration distribution of the pool of 10 000 structures generated by EOM (dotted lines) and the R_g_ distribution of the selected ensembles (solid lines). (**C**) The ensemble of 50 BR_187–385_ molecules selected by EOM was separated into two groups. The first group had an R_g_ value of around 20 Å and is represented by the molecular model with the extension colored in cyan. The second had an extended R_g_ value of 22.5 Å and is represented by the molecular model with the extension colored in blue. (**D**) The ensemble of 50 BR*_120–395_ molecules had a similar distribution of R_g_ values as compared the large pool of molecules with random conformations of the extensions. The two illustrated models displayed in blue and cyan have R_g_ values of 52 and 26 Å, respectively.

In order to obtain structural information about the longer BR*_120–395_ construct in solution, comparative small angle X-ray scattering (SAXS) data were collected for both monomeric BR_187–385_ and BR*_120–395_ (Fig. 2A,B and Table S1). The directly accessible and sample-characteristic radii of gyration (R_g_) and maximal dimensions (D_max_) of BR_187–385_ and BR_120–395_ were 20 ± 1 Å and 29 ± 2 Å as well as 78 ± 8 Å and 125 ± 12 Å, respectively, indicating a more extended structure for BR_120–395_ (Fig. S3A,B). Indeed, molecular models obtained from the ensemble optimization method (EOM) support the presence of a single globular domain common to BR_187–385_ and BR*_120–395_, but with a longer highly mobile structural extension for BR_120–395_ (Fig. 2C,D). In dimensionless Kratky plots, the curve of BR_187–385_ closely resembles the bell-shaped curve expected for a globular protein, while the curve of BR_120–395_ is clearly shifted towards the shape of a molecule that comprises a more disordered, random chain (Fig. S3C). This is in line with our interpretation that the longer BR_120–395_ construct comprises an extended, highly flexible section in solution compared to BR_187–385_. Additional structural information was obtained from circular dichroism spectra of BR_187–385_ and BR*_120–395_ that also indicated the presence of disordered and helical regions for the additional residues in BR*_120–395_ (Fig. S3D). Combining the structural information obtained from AUC, SAXS, and CD, we conclude that the N-terminal region in BR*_120–395_ does not promote aggregation and corresponds to a non-globular, largely disordered structure.

### BR_187–385_ forms low-affinity homo-dimers through intermolecular β-sheets

**Figure 3:**
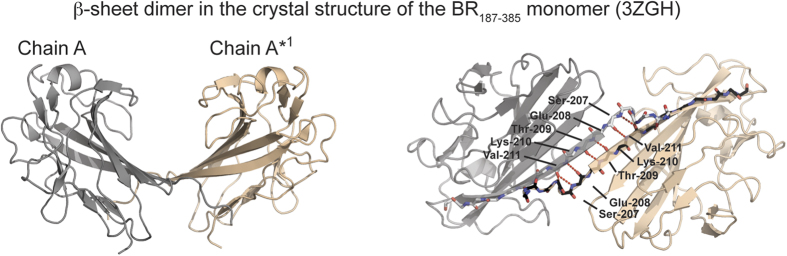
BR_187–385_ forms a β-sheet dimer with a saddle-like structure. Intermolecular β-sheets are formed between symmetry mates of BR_187–385_ in the P4_1_22 (PDB: 3ZGH) crystal structure with a total interface surface area (ISA) of 600 Å^2^. The sheet is stabilized by eight hydrogen bonds between the backbones of the two A1 strands. The molecules forming intermolecular β-sheets are colored grey and wheat. The view of the right panel was rotated by ~90° compared to the left panel. Hydrogen bond interactions are represented as dashed red lines.

Considering that the described domain swap mechanism is unlikely to occur spontaneously *in vivo*, and that the previously suggested self-aggregating BR_120–166_ region does not promote self-oligomerization in our experiments, we looked back into the crystal structure of the isolated BR_187–385_ monomer^20^ for hints of a more probable oligomerization mechanism. Indeed, we discovered intermolecular β-sheets between symmetry-related monomeric molecules in the P4_1_22 crystal form (PDB: 3ZGH) and in the P4_3_2_1_2 crystal form (PDB: 3ZGI) forming dimers with interface surface areas of 600 Å^2^ and 440 Å^2^, respectively (Figs 3 and S4A). Moreover, intermolecular β-sheets are also observed between symmetry-related domain-swapped dimers with similar interfaces and hydrogen bond patterns (Fig. S4B,C). However, although the formation of such edge-to-edge β-sheets is regarded as a fundamental feature underlying molecular recognition in protein-protein interactions^34^, a predicted weak interaction of this interface could prevent its detection in the performed SEC, SAXS and AUC experiments (Figs 1C and 2A and S1B)^20^.

Single-cell and single-molecule atomic force microscopy have recently been applied to demonstrate that low-affinity (15 mM) homophilic interactions of the fibronectin binding protein A (FnBPA) of *Staphylococcus aureus* promote cellular aggregation^28^. Therefore, we also applied single-molecule force spectroscopy to test the capacity of BR_187–378_ and BR*_120–395_ to mediate similar low-affinity homophilic bonds. Indeed, homophilic binding events were measured for BR_187–378_ and BR*_120–395_ with mean adhesion forces of 70 ± 18 pN and 68 ± 14 pN, respectively (Fig. S5A–C). And as expected for specific bimolecular bonds^35^, the adhesion forces increased linearly with the logarithm of the loading rate (Fig. S5D).

### BR_187–385_ binds to DNA and forms a positively charged saddle that snuggly fits the acidic helical structure of double-stranded DNA

**Figure 4:**
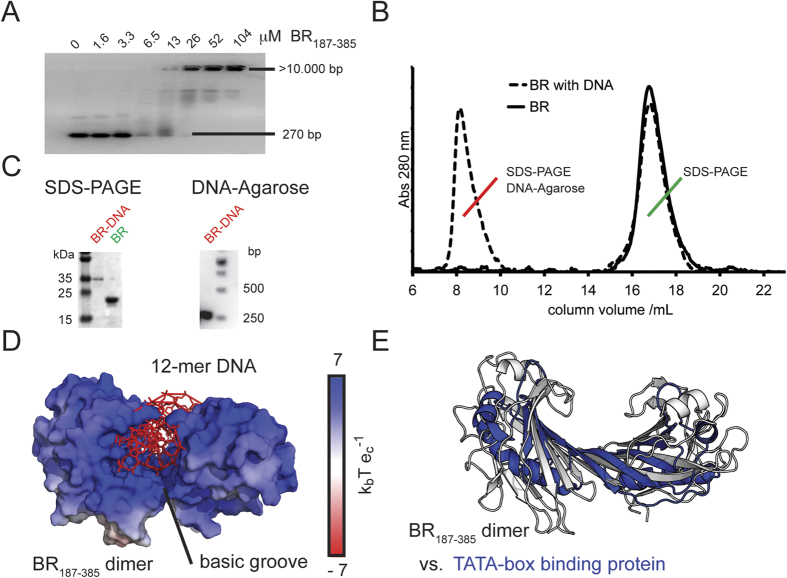
Basic BR_187–385_ binds to DNA and forms a saddle-like dimer structure that snuggly fits the acidic double stranded DNA helix. (**A**) BR_187–385_ concentration-dependent complex formation with a non-specific 270 bp DNA molecule (DNA_270bp_) was detected as retarded DNA bands in an electrophoretic mobility shift assay (EMSA). The apparent affinity of the interaction was estimated to around 10 μM. (**B**) After incubation of BR_187–385_ with DNA_270bp_, the area of the BR_187–385_ monomer peak, obtained at a retention volume of around 17 mL and an absorption wavelength of 280 nm, was slightly reduced. The high-molecular weight population was detected at a retention volume of about 8 mL with an estimated MW of about >1 MDa. DNA_270bp_ alone would elute at the same retention volume (not shown). (**C**) The putative BR_187–385_-DNA complex was isolated from SEC and run on SDS-PAGE (left) and DNA-Agarose (right) gels. In SDS-PAGE the protein band was detected as a slightly larger complex, possibly retarded by the bound negatively charged DNA. The DNA_270bp_ that was isolated from SEC was detected at the expected size, and was not retarded due to the low amount of bound BR_187–385_. (**D**) The intermolecular β-sheet dimer resembles a saddle-like structure with an extended intermolecular β-sheet. The electrostatic potentials are plotted in k_b_T e_c_^−1^ with the Boltzmann’s constant k_b_, the charge of an electron e_c_ at a temperature T of 298 K. The saddle-like structure snuggly fits an acidic helical non-specific DNA molecule, as predicted in a molecular model of the BR_187–385_-DNA complex. (**E**) Similar to the BR_187–385_ dimer, the TATA-box binding protein (TBP, PDB: 1YTB) adopts a saddle-like structure. TBP was superimposed onto the BR_187–387_ dimer via shape similarity and represented as white and blue ribbons.

Although the measured weak homophilic binding forces could be sufficient for cellular aggregation, we hypothesized that the highly basic BR_187–385_ could also bind to acidic biofilm-associated extracellular DNA. Indeed, electromobility shift assays (EMSA) demonstrated binding of a randomly chosen 276 bp long DNA (DNA_276bp_) molecule to BR_187–385_ with an apparent affinity in the lower micromolar range (Fig. 4A). After incubation of BR_187–378_ with DNA_276bp_ at a 1:750 molar ratio, the purified high-molecular weight DNA population also comprised bound BR_187–385_ molecules (Fig. 4B,C). Interestingly, the DNA-bound BR_187–385_ ran at a higher molecular weight in SDS-PAGE, indicating that the DNA was not completely removed even by lauryl-dodecyl sulfate detergent treatment. Based on our previously suggested ligand-binding model, it is tempting to speculate that the negatively charged DNA binds to the extended highly basic groove of the intermolecular β-sheet dimer of BR_187–385_ (Fig. 4D). Molecular dynamic (MD) simulations also suggested that the docked DNA:BR_187–385_ complex is flexible but most of the non-nucleotide specific protein-DNA contacts are preserved over a 50 ns simulation period (Movie S1).

Strikingly, the structure of the BR_187–385_ dimer resembles the molecular saddle of the transcriptional pre-initiation complex-associated TATA-box binding protein (TBP) (Fig. 4E). While the concave under-surface of the TBP saddle is also highly basic, the specificity of the protein:TATATAAA interaction is primarily mediated through hydrophobic interactions^36^,^37^. However, it is well established that the specificity of protein-DNA interactions is manifested entirely in the non-electrostatic interaction component, while the electrostatic component typically contributes to the majority of the affinity^38^. Similar to the interaction of TBP with non-related DNA, the apparent affinity measured for binding of BR_187–385_ to DNA was in the low micromolar range. Thus, although we cannot exclude the possibility that PsrP recognizes a specific DNA sequence, we here hypothesize that PsrP binds non-specifically to DNA.

### BR_187–385_ promotes DNA-dependent bacterial aggregation

**Figure 5:**
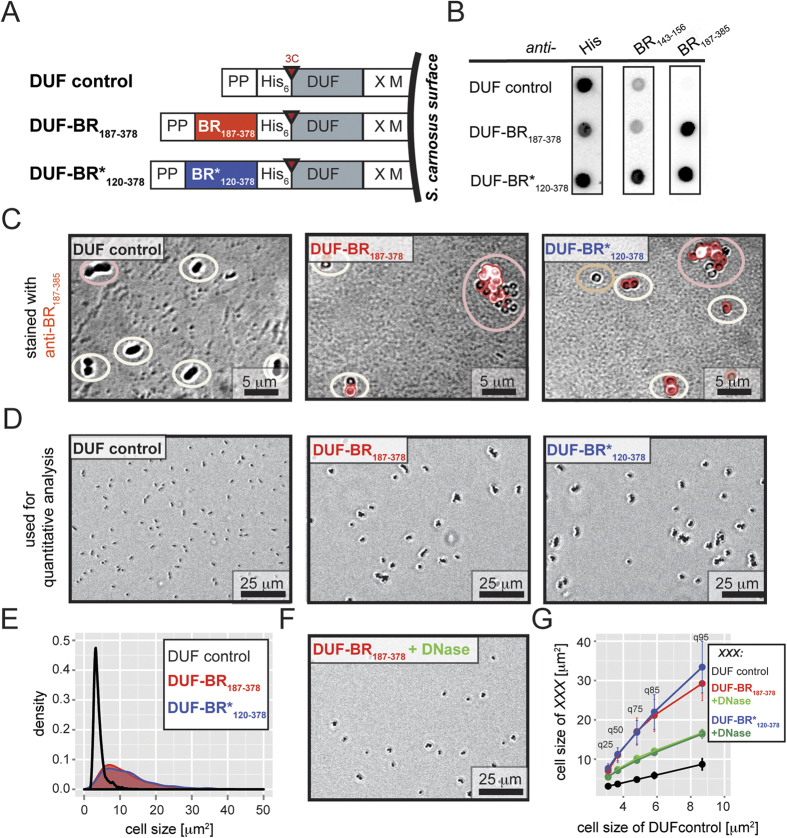
Cell-surface display of BR_187–385_ and BR*_120–378_ promotes eDNA-dependent bacterial aggregation of *Staphylococcus carnosus*. (**A**) The domain organization for surface display of BR_187–378_ and BR*_120–378_ on the surface of *S. carnosus.* PP - propeptide from *S. hyicus* lipase; His_6_ – hexahistidine tag; 3C - recognition sequence for protease 3C; X and M - regions from the SpA gene for covalent anchoring of the proteins to the peptidoglycan cell wall. (**B**) Supernatants of 3C-protease incubated staphylococcal cells were spotted on nitrocellulose membranes. Staining using polyclonal anti-serum against BR_187–385_ confirmed the expression of both BR*_120–378_ and BR_187–378_, and the absence and presence of signal for the BR_143–156_ region allowed for an unambiguous discrimination between BR*_120–378_- and BR_187–378_-expressing cells, respectively. As expected, signals from HRP-coupled anti-His antibodies were detected for all three constructs. (**C**) The BR_187–378_ and BR*_120–378_ displaying cells were stained using fluorescently labeled secondary antibodies directed against primary polyclonals against BR_187–385_. Singlets, duplets and higher aggregates are highlighted in white, bronze and pink, respectively. (**D**) Phase-like contrast microscopy images revealed the formation of cellular aggregates of *S. carnosus* displaying BR_187–378_ and BR*_120–378_, but not of cells displaying the DUF domain alone. (**E**) The two-dimensional aggregates sizes of staphylococcal cells were quantitatively analyzed from an area of about 0.05 mm^2^, which corresponds to approximately 64 times the area shown in panel D. The derived particle areas are plotted as normalized density histogram distributions, with the Scar-DUF control and the corresponding samples in black and red, respectively. The histogram represents the particle distribution obtained from a single experiment. The results from three independent experiments are summarized in the q-q plot shown in panel G. (**F**) Incubation of Scar-BR_187–378_ with DNaseI reduced the formation of aggregates, compared to the same sample not incubated with DNaseI (panel D). (**G**) The mean particle area sizes of quantiles with cut-off values at 25%, 50%, 75%, 85%, and 95% were calculated from three independent experiments, and summarized in a quantile-quantile plot (q-q plot). After incubation with DNaseI, both q-q plot curves of Scar-BR_187–378_ and Scar-BR_120–378_ are downward-shifted (light and dark green, respectively).

Display of heterologously produced proteins on the surface of the Gram-positive bacterium *Staphylococcus carnosus* represents an excellent model system to study the functions of surface-associated proteins, *e.g.* the biofilm-promoting function of the SasC protein of *Staphylococcus aureus*^39^,^40^. In order to assess the capacity of each segment of the BR domain to promote cellular aggregation, BR_187–378_ and BR*_120–378_ were fused separately to the SasC-derived DUF-domain linker followed by a C-terminal LPxTG motif that becomes covalently attached to the staphylococcal cell wall through Sortase-mediated enzymatic linkage (Fig. 5A). The DUF domain was chosen to substitute the role of the SRR domain of native PsrP, which is to extend the BR region away from the cellular surface for functional accessibility. Dotblot assays of 3C-protease-cleaved surface-released proteins using polyclonal antisera against BR_143–156_ and BR_187–378_ confirmed that both BR*_120–378_ and BR_187–378_ were produced (Fig. 5B). The poly-His tags were detected for all constructs including the DUF control strain.

While bacteria displaying the DUF linker domain alone (Scar-DUF) were mainly observed as single cells or in doublets, cells displaying either BR*_120–378_ (Scar-BR*_120–378_) or BR_187–378_ (Scar-BR_187–378_) were heavily aggregated (Fig. 5C,D). Using fluorescence phase-contrast microscopy, single bacteria of Scar-BR_187–378_ and -BR*_120–378_ could be easily discriminated even within aggregates, while this was more difficult for Scar-DUF that appeared mostly in duplets (Fig. 5C). The origin of the slightly altered morphology of Scar-DUF bacteria under phase-contrast compared to the Scar-BR constructs is not known, but could be related to the absence and presence of the positively charged BR proteins, respectively. While Scar-DUF was not stained when incubated with fluorescently labeled secondary antibodies directed against primary polyclonals against BR_187–385_, both Scar-BR*_120–378_ and Scar-BR_187–378_ bacteria were stained (Fig. 5C).

Quantitative analysis of the collected microscopy images confirmed our initial qualitative estimation that Scar-BR_187–378_ and Scar-BR*_120–378_ were significantly more aggregated compared to Scar-DUF, which is apparent from the particle size histogram plots with median particle sizes (q50) of 11 ± 2 μm^2^, 11 ± 2 μm^2^ and 3.7 ± 0.2 μm^2^, respectively (Fig. 5D,E). Since the staphylococcal cells were grown in terrific broth (TB) medium comprising yeast extract, we reasoned that the DNA extracted from yeast contributed to the observed cellular aggregates. Indeed, following incubation with the DNA-degrading enzyme DNaseI, aggregates of Scar-BR*_120–378_ and Scar-BR_187–378_ were reduced by almost 40% over all listed quantile values (Fig. 5F,G). A quantile is the fraction at which x% percent of the data fall below and (100 − x)% fall above that value. While two identical populations give a straight line (Fig. 5G, Scar-DUF in black), upward-shifted curves reveal increased particle sizes for Scar-BR_187–378_ and Scar-BR_120–378_ (Fig. 5G, Scar-BR_187–378_ and Scar-BR_120–378_ in red and blue, respectively). Similar results were obtained when Scar-DUF, Scar-BR_187–378_ and Scar-BR_120–378_ were grown in M9 minimal medium in the presence and absence of added sheared eDNA derived from herring sperm (Fig. S6). While presence of eDNA did not have any effect on Scar-DUF, both Scar-BR_187–378_ and Scar-BR_120–378_ were significantly more aggregated, albeit at a slightly reduced extent compared to bacteria grown in TB. We hypothesize that the observed slight reduction can be attributed to the restricted sequence length of the added eDNA between 15 and 50 bp. From these experiments we conclude that the globular BR_187–378_ domain of PsrP also promotes eDNA-dependent cellular aggregation within pneumococcal biofilms. Future experiments will address the exact contribution of each of the two factors, namely BR dimerization and binding to eDNA for biofilm formation.

## Conclusions

PsrP has previously been suggested to promote pneumococcal biofilm formation through residues 122–166 (BR_122–166_), preceding the globular KRT10-binding MSCRAMM-related BR_187–385_ domain. In this study, small angle X-ray scattering and circular dichroism spectroscopy analysis revealed a non-globular, disordered structure for the BR_122–166_ region that does not promote self-aggregation. Importantly, surface-display of BR_187–385_ demonstrated the capacity of the globular domain to promote eDNA-dependent bacterial aggregation. Structural studies indicate that the saddle-like structure of the BR_187–385_ β-sheet dimer snuggly fits the acidic helical double-stranded DNA structure.

## Materials and Methods

### Cloning, expression and purification of protein constructs

Expression constructs comprising residues 120–395 of PsrP (BR_120–395_), 187–385 (BR_187–385_), and 187–378 (BR_187–378_) all with a N-terminal poly-His (HHHHHH) tag, were cloned into the pET21d (Novagen) expression vector as described previously^20^. The mutated expression construct of BR_120–395_ (BR*_120–395_) with R165S and R168S substitutions was generated following previously described protocols^41^. All coding sequences of the protein-expression constructs were confirmed by DNA sequencing and are listed in the supplemental information.

Heterologous protein expression in *E. coli* (T7 express, New England Biolabs) was induced at OD 0.4–0.7 using 400 μM IPTG and performed over night at 25 °C. The poly-Histidine-tagged proteins were purified using immobilized metal affinity (IMAC), cation exchange chromatography (CEC) (HisTrap FF and HiTrap SPFF, GE-Healthcare) and size exclusion chromatography (SEC) on Superdex 75 or 200 columns (GE Healthcare).

### Furin cleavage assay

Two units of Furin protease (New England Biolabs, P8077) were added to a total assay volume of 100 μL with a concentration of 0.7 mg/mL BR_120–395_ and mutated BR*_120–395_ in 20 mM HEPES, 500 mM NaCl, 10% glycerol, 1 mM CaCl_2_, pH 7.5. Hexa-D-arginine amide (Sigma: SCP0148) was used as furin inhibitor at concentrations of 100 μM and 250 μM. The cleavage assays were carried out at room temperature and at 37 °C. Samples for SDS-PAGE were taken at after 0 h, 2 h, 4 h and overnight incubation.

### Small angle X-ray scattering

#### Sample preparation and data collection

Data were collected at beamline BM29 at the European Synchrotron Radiation Facility (ESRF, Grenoble)^42^. Using a Pilatus 1 M detector at a sample to detector distance of 2.9 m and a wavelength of λ = 0.9919 Å, the range of momentum transfer 0.006 < s < 0.45 Å^−1^ was covered (s = 4π sin θ/λ, where 2θ is the scattering angle). Prior to data collection, proteins were buffer exchanged into phosphate buffered saline (PBS) with 5% glycerol, pH 7.4. Samples were measured at concentrations between 0.3 and 3 mg/ml using a continuous flow cell capillary.

#### Data analysis

The forward scattering I(0), the radius of gyration R_g_ along with the pair distribution function of the particle p(r) and the maximum dimension D_max_ were derived using the automated SAXS data analysis pipeline^43^. Using Ensemble Optimization Method (EOM)^44^ analysis of BR_187–385_ and BR_120–395_, a pool of 10,000 models comprising a rigid domain (residues K206-E375) and flexible N- and C-termini were generated. A subset of the pool was selected using a genetic algorithm such that the calculated averaged scattering of the selected models agreed with the experimental data. The R_g_ distributions of the selected ensembles were obtained by repeating the selection process multiple times.

### Structural analysis

All figures were created using PyMOL version 1.3.0^45^. Interfaces of macromolecular assemblies in the crystal structures were investigated using PBDePISA^46^. The molecular model of the BR_187–385_-DNA complex was predicted using the non-specific DNA-rigid protein-docking algorithm ParaDock^47^, and the electrostatic surface was visualized using APBS^48^,^49^. The structure of TBP (PDB: 1YTB) was superimposed onto the BR_187–387_ dimer using the shape similarity algorithm of subcomp in which three-dimensional models are represented as simplified ensembles of points^36^,^50^.

### Electrophoretic mobility shift assay (EMSA) and isolation of BR_187–378_-DNA_276bp_ complex using SEC

The 276 bp DNA molecule (DNA_276_ sequence listed in supplemental information) was produced and isolated by PCR amplification and gel extraction (QIAEXII gel extraction kit, Qiagen). BR_187–385_ was titrated to DNA at a final concentration of 100 nM in TAE buffer (40 mM Tris, 20 mM acetic acid, and 1 mM EDTA) supplemented with 300 mM NaCl. After an hour incubation time, the samples were mixed with sample loading buffer and run on a 1.5% (w/V) GelGreen-stained (Biotium) agarose gel in TAE buffer.

For SEC analysis, BR_187–385_ and DNA_276_ were incubated at concentrations of 30 μM and 440 nM, respectively. Before mixing, BR_187–385_ and DNA_276_ were prepared in 20 mM Hepes, 200 mM NaCl, 10% glycerol, pH 7.5 and 10 mM Tris at pH 8.5, respectively. The mixed samples were loaded on a Superdex S200 10/300 GL (GE healthcare) equilibrated in 20 mM Hepes, 200 mM NaCl, 10% glycerol, pH 7.5.

### Surface display of BR on the surface of *Staphylococcus carnosus*

#### Cellular aggregation assay

The PCR-amplified PsrP constructs and the gene-synthesized (Eurofins Genomics, Germany) DUF1542 repeat domain of SasC (Uniprot ID: C7BUR8) were cloned into the staphylococcal display vector ‘pHis3C’ using a sequence and ligation independent cloning (SLIC) method^51^,^52^. Coding sequences of the protein-display constructs were confirmed by DNA sequencing and are listed in the supplemental information. Transformed *S. carnosus* cells were cultured in TB medium with chloramphenicol (10 ug/mL). OD-adjusted cells treated or non-treated with DNaseI were gently re-suspended in PBS, filled into wells of a 96-μ-well plate (Ibidi, Germanu) and images were taken using the ZOE Fluorescent Cell Imager (Biorad, USA) at 20x magnification. Bacteria were also grown in M9 derived minimal medium^53^ comprising 1x M9 salts, 2 mM MgSO_4_, 0.1 mM CaCl_2_, 1% glucose, 1% casaminoacids, 1 mM Thiamine-HCl and 0.05 mM nicotinamide, with or without the addition of herring sperm derived degraded DNA (D3159, Sigma Aldrich) at a concentration of 10 ug/mL that corresponds to approximately 1 μM of 15 bp oligonucleotide fragments. The bacteria were adjusted in OD and imaged in the same way as described previously. Images were analyzed using the software cell profiler (www.cellprofiler.org), the particle parameter values were imported into the R software package for statistical analysis and visualization using density histogram and q-q plots^54^,^55^.

#### Dotblot assays and fluorescence imaging

Polyclonal antibodies against the TEV-cleaved purified BR_187–385_ monomer and a synthesized BR_143–156_ (RKKPASDYVASVTN) peptide were raised in rabbits and the obtained sera yielded titers of about 75000 and 6000, respectively (Innovagen, Sweden). For fluorescent staining, bacteria were incubated with affinity-purified anti-BR_187–385_ antibodies and detected using fluorescein isothiocyanate (FITC)-labeled goat anti-rabbit IgG (life technologies) secondary antibody. Bacteria were fixed in 4% paraformaldehyde, washed, and visualized using a fluorescence microscope (Leica Leitz DMRBE). For dotblot assays, surface-displayed proteins were cleaved as previously described^52^ and applied on nitrocellulose filter membranes. Proteins were detected using polyclonal rabbit-antisera against BR_187–385_, BR_143–156_ as well as HRP-coupled anti-His antibodies (ab1187; Abcam, UK). The anti-BR antibody stained samples were incubated using HRP-coupled monoclonal anti-rabbit IgGγ (A1949, Sigma Aldrich). Membranes were developed using Pierce ECL western blotting substrate (Thermo Scientific). Further details are given in the supplementary material.

## Additional Information

**Accession codes**: Coordinates and structure factors have been deposited in the Protein Data Bank with accession number 5JUI. SAXS data have been deposited in the Small Angle Scattering Biological Databank (sasbdb) with accession numbers SASDAC6 and SASDAE6.

**How to cite this article**: Schulte, T. *et al*. The BR domain of PsrP interacts with extracellular DNA to promote bacterial aggregation; structural insights into pneumococcal biofilm formation. *Sci. Rep.* **6**, 32371; doi: 10.1038/srep32371 (2016).

## Supplementary Material

Supplementary Information

Supplementary Movie S1

## References

[b1] van der Poll T. & Opal S. M. Pathogenesis, treatment, and prevention of pneumococcal pneumonia. Lancet 374, 1543–1556 (2009). 19880020 10.1016/S0140-6736(09)61114-4

[b2] Costerton J. W., Stewart P. S. & Greenberg E. P. Bacterial Biofilms: A Common Cause of Persistent Infections. Science 284, 1318–1322 (1999). 10334980 10.1126/science.284.5418.1318

[b3] Hall-Stoodley L., Costerton J. W. & Stoodley P. Bacterial biofilms: from the Natural environment to infectious diseases. Nat. Rev. Microbiol. 2, 95–108 (2004). 15040259 10.1038/nrmicro821

[b4] Simell B. . The fundamental link between pneumococcal carriage and disease. Expert Rev. Vaccines 11, 841–855 (2012). 22913260 10.1586/erv.12.53

[b5] Domenech M., Garcia E. & Moscoso M. Biofilm formation in *Streptococcus pneumoniae*. Microb. Biotechnol. 5, 455–465 (2012). 21906265 10.1111/j.1751-7915.2011.00294.x PMC3815323

[b6] Domenech M., García E., Prieto A. & Moscoso M. Insight into the composition of the intercellular matrix of *Streptococcus pneumoniae* biofilms. Environ. Microbiol. 15, 502–516 (2013). 22913814 10.1111/j.1462-2920.2012.02853.x

[b7] Blanchette-Cain K. . *Streptococcus pneumoniae* Biofilm Formation is Strain Dependent, Multifactorial, and Associated with Reduced Invasiveness and Immunoreactivity during Colonization. mBio 4, e00745-13 (2013). 10.1128/mBio.00745-13 PMC3812715 24129258

[b8] Hobley L., Harkins C., MacPhee C. E. & Stanley-Wall N. R. Giving structure to the biofilm matrix: an overview of individual strategies and emerging common themes. FEMS Microbiol. Rev. fuv015, 10.1093/femsre/fuv015 (2015). PMC4551309 25907113

[b9] Flemming H.-C. & Wingender J. The biofilm matrix. Nat. Rev. Microbiol. 8, 623–633 (2010). 20676145 10.1038/nrmicro2415

[b10] van Schaik E. J. . DNA binding: a novel function of *Pseudomonas aeruginosa* type IV pili. J. Bacteriol. 187, 1455–1464 (2005). 15687210 10.1128/JB.187.4.1455-1464.2005 PMC545619

[b11] Camilli R., Pantosti A. & Baldassarri L. Contribution of serotype and genetic background to biofilm formation by *Streptococcus pneumoniae*. Eur. J. Clin. Microbiol. Infect. Dis. Off. Publ. Eur. Soc. Clin. Microbiol. 30, 97–102 (2011). 10.1007/s10096-010-1060-6 20844912

[b12] Hall-Stoodley L. . Characterization of biofilm matrix, degradation by DNase treatment and evidence of capsule downregulation in *Streptococcus pneumoniae* clinical isolates. BMC Microbiol. 8, 173 (2008). 18842140 10.1186/1471-2180-8-173 PMC2600794

[b13] Sanchez C. J. . Streptococcus pneumoniae in Biofilms Are Unable to Cause Invasive Disease Due to Altered Virulence Determinant Production. Plos One 6, e28738 (2011). 22174882 10.1371/journal.pone.0028738 PMC3234282

[b14] Muñoz-Elías E. J., Marcano J. & Camilli A. Isolation of *Streptococcus pneumoniae* Biofilm Mutants and Their Characterization during Nasopharyngeal Colonization. Infect. Immun. 76, 5049–5061 (2008). 18794289 10.1128/IAI.00425-08 PMC2573321

[b15] Sanchez C. J. . The pneumococcal serine-rich repeat protein is an intra-species bacterial adhesin that promotes bacterial aggregation *in vivo* and in biofilms. Plos Pathog. 6 (2010). 10.1371/journal.ppat.1001044 PMC2920850 20714350

[b16] Ramboarina S. . Structural insights into serine-rich fimbriae from gram-positive bacteria. J. Biol. Chem. 285, 32446–32457 (2010). 20584910 10.1074/jbc.M110.128165 PMC2952246

[b17] Pyburn T. M. . A Structural Model for Binding of the Serine-Rich Repeat Adhesin GspB to Host Carbohydrate Receptors. Plos Pathog. 7, e1002112 (2011). 21765814 10.1371/journal.ppat.1002112 PMC3131266

[b18] Lizcano A., Sanchez C. J. & Orihuela C. J. A role for glycosylated serine-rich repeat proteins in Gram-positive bacterial pathogenesis. Mol. Oral Microbiol. 27, 257–269 (2012). 22759311 10.1111/j.2041-1014.2012.00653.x PMC3390760

[b19] Shivshankar P., Sanchez C., Rose L. F. & Orihuela C. J. The *Streptococcus pneumoniae* adhesin PsrP binds to Keratin 10 on lung cells. Mol. Microbiol. 73, 663–679 (2009). 19627498 10.1111/j.1365-2958.2009.06796.x PMC2753542

[b20] Schulte T. . The basic keratin 10-binding domain of the virulence-associated pneumococcal serine-rich protein PsrP adopts a novel MSCRAMM fold. Open Biol. 4, 130090 (2014). 24430336 10.1098/rsob.130090 PMC3909270

[b21] Garnett J. A. . Structural insight into the role of *Streptococcus parasanguinis* Fap1 within oral biofilm formation. Biochem. Biophys. Res. Commun. 417, 421–426 (2012). 22166217 10.1016/j.bbrc.2011.11.131 PMC3518267

[b22] Wu H., Zeng M. & Fives-Taylor P. The glycan moieties and the N-terminal polypeptide backbone of a fimbria-associated adhesin, Fap1, play distinct roles in the biofilm development of *Streptococcus parasanguinis*. Infect. Immun. 75, 2181–2188 (2007). 17296746 10.1128/IAI.01544-06 PMC1865748

[b23] Garnett J. A. & Matthews S. Interactions in Bacterial Biofilm Development: A Structural Perspective. Curr. Protein Pept. Sci. 13, 739–755 (2012). 23305361 10.2174/138920312804871166 PMC3601411

[b24] Corrigan R. M., Rigby D., Handley P. & Foster T. J. The role of *Staphylococcus aureus* surface protein SasG in adherence and biofilm formation. Microbiology 153, 2435–2446 (2007). 17660408 10.1099/mic.0.2007/006676-0

[b25] Gruszka D. T. . Staphylococcal Biofilm-Forming Protein Has a Contiguous Rod-Like Structure. Proc. Natl. Acad. Sci. 109, E1011–E1018 (2012). 22493247 10.1073/pnas.1119456109 PMC3340054

[b26] Rohde H. . Induction of *Staphylococcus epidermidis* biofilm formation via proteolytic processing of the accumulation-associated protein by staphylococcal and host proteases. Mol. Microbiol. 55, 1883–1895 (2005). 15752207 10.1111/j.1365-2958.2005.04515.x

[b27] Conrady D. G., Wilson J. J. & Herr A. B. Structural basis for Zn^2+^-dependent intercellular adhesion in staphylococcal biofilms. Proc. Natl. Acad. Sci. USA 110, E202–211 (2013). 23277549 10.1073/pnas.1208134110 PMC3549106

[b28] Herman-Bausier P., El-Kirat-Chatel S., Foster T. J., Geoghegan J. A. & Dufrêne Y. F. Staphylococcus aureus Fibronectin-Binding Protein A Mediates Cell-Cell Adhesion through Low-Affinity Homophilic Bonds. mBio 6 (2015). 10.1128/mBio.00413-15 PMC4447249 26015495

[b29] Klimpel K. R., Molloy S. S., Thomas G. & Leppla S. H. Anthrax toxin protective antigen is activated by a cell surface protease with the sequence specificity and catalytic properties of furin. Proc. Natl. Acad. Sci. 89, 10277–10281 (1992). 1438214 10.1073/pnas.89.21.10277 PMC50321

[b30] Thomas G. Furin at the cutting edge: From protein traffic to embryogenesis and disease. Nat. Rev. Mol. Cell Biol. 3, 753–766 (2002). 12360192 10.1038/nrm934 PMC1964754

[b31] Karunanithi S. . Shedding of the Mucin-Like Flocculin Flo11p Reveals a New Aspect of Fungal Adhesion Regulation. Curr. Biol. 20, 1389–1395 (2010). 20619652 10.1016/j.cub.2010.06.033 PMC2918736

[b32] Gronenborn A. M. Protein acrobatics in pairs–dimerization via domain swapping. Curr. Opin. Struct. Biol. 19, 39–49 (2009). 19162470 10.1016/j.sbi.2008.12.002 PMC2676429

[b33] Bennett M. J. & Eisenberg D. Refined structure of monomeric diphtheria toxin at 2.3 A resolution. Protein Sci. Publ. Protein Soc. 3, 1464–1475 (1994). 10.1002/pro.5560030912 PMC2142954 7833808

[b34] Nowick J. S. & Chung D. M. Sequence-Selective Molecular Recognition between β Sheets. Angew. Chem. Int. Ed. 42, 1765–1768 (2003). 10.1002/anie.200250750 12707901

[b35] Hinterdorfer P. & Dufrêne Y. F. Detection and localization of single molecular recognition events using atomic force microscopy. Nat. Methods 3, 347–355 (2006). 16628204 10.1038/nmeth871

[b36] Kim Y., Geiger J. H., Hahn S. & Sigler P. B. Crystal structure of a yeast TBP/TATA-box complex. Nature 365, 512–520 (1993). 8413604 10.1038/365512a0

[b37] Hahn S., Buratowski S., Sharp P. A. & Guarente L. Yeast TATA-binding protein TFIID binds to TATA elements with both consensus and nonconsensus DNA sequences. Proc. Natl. Acad. Sci. USA 86, 5718–5722 (1989). 2569738 10.1073/pnas.86.15.5718 PMC297701

[b38] Privalov P. L., Dragan A. I. & Crane-Robinson C. Interpreting protein/DNA interactions: distinguishing specific from non-specific and electrostatic from non-electrostatic components. Nucleic Acids Res. 39, 2483–2491 (2011). 21071403 10.1093/nar/gkq984 PMC3074165

[b39] Samuelson P., Gunneriusson E., Nygren P. A. & Ståhl S. Display of proteins on bacteria. J. Biotechnol. 96, 129–154 (2002). 12039531 10.1016/s0168-1656(02)00043-3

[b40] Schroeder K. . Molecular Characterization of a Novel *Staphylococcus Aureus* Surface Protein (SasC) Involved in Cell Aggregation and Biofilm Accumulation. Plos One 4, e7567 (2009). 19851500 10.1371/journal.pone.0007567 PMC2761602

[b41] Li J. . Site-directed mutagenesis by combination of homologous recombination and DpnI digestion of the plasmid template in *Escherichia coli*. Anal. Biochem. 373, 389–391 (2008). 18037368 10.1016/j.ab.2007.10.034

[b42] Pernot P. . Upgraded ESRF BM29 beamline for SAXS on macromolecules in solution. J. Synchrotron Radiat. 20, 660–664 (2013). 23765312 10.1107/S0909049513010431 PMC3943554

[b43] Franke D., Kikhney A. G. & Svergun D. I. Automated acquisition and analysis of small angle X-ray scattering data. Nucl. Instrum. Methods Phys. Res. Sect. Accel. Spectrometers Detect. Assoc. Equip. 689, 52–59 (2012).

[b44] Bernadó P., Mylonas E., Petoukhov M. V., Blackledge M. & Svergun D. I. Structural characterization of flexible proteins using small-angle X-ray scattering. J. Am. Chem. Soc. 129, 5656–5664 (2007). 17411046 10.1021/ja069124n

[b45] Schrödinger L. *PyMOL Molecular Graphics System*. (2010).

[b46] Krissinel E. & Henrick K. Inference of macromolecular assemblies from crystalline state. J. Mol. Biol. 372, 774–797 (2007). 17681537 10.1016/j.jmb.2007.05.022

[b47] Banitt I. & Wolfson H. J. ParaDock: a flexible non-specific DNA—rigid protein docking algorithm. Nucleic Acids Res. 39, e135 (2011). 21835777 10.1093/nar/gkr620 PMC3203577

[b48] Dolinsky T. J., Nielsen J. E., McCammon J. A. & Baker N. A. PDB2PQR: an automated pipeline for the setup of Poisson-Boltzmann electrostatics calculations. Nucleic Acids Res. 32, W665–7 (2004). 15215472 10.1093/nar/gkh381 PMC441519

[b49] Baker N. A., Sept D., Joseph S., Holst M. J. & McCammon J. A. Electrostatics of nanosystems: application to microtubules and the ribosome. Proc. Natl. Acad. Sci. USA 98, 10037–41 (2001). 11517324 10.1073/pnas.181342398 PMC56910

[b50] Kozin M. B. & Svergun D. I. Automated matching of high- and low-resolution structural models. J. Appl. Crystallogr. 34, 33–41 (2001).

[b51] Li M. Z. & Elledge S. J. Harnessing homologous recombination *in vitro* to generate recombinant DNA via SLIC. Nat. Methods 4, 251–256 (2007). 17293868 10.1038/nmeth1010

[b52] Kronqvist N., Löfblom J., Severa D., Ståhl S. & Wernérus H. Simplified characterization through site-specific protease-mediated release of affinity proteins selected by staphylococcal display. FEMS Microbiol. Lett. 278, 128–136 (2008). 18034830 10.1111/j.1574-6968.2007.00990.x

[b53] Reed P. . Staphylococcus aureus Survives with a Minimal Peptidoglycan Synthesis Machine but Sacrifices Virulence and Antibiotic Resistance. Plos Pathog. 11, e1004891 (2015). 25951442 10.1371/journal.ppat.1004891 PMC4423922

[b54] Jones T. R. . CellProfiler Analyst: data exploration and analysis software for complex image-based screens. BMC Bioinformatics 9, 482 (2008). 19014601 10.1186/1471-2105-9-482 PMC2614436

[b55] R Core Team. R: A language and environment for statistical computing. (R Foundation for Statistical Computing) (2011).

