# Supplementary material for: The BR domain of PsrP interacts with extracellular DNA to promote bacterial aggregation; structural insights into pneumococcal biofilm formation
Source: Sci Rep. 2016 Sep 1;6:32371. doi: 10.1038/srep32371 (PMC5007671; doi:10.1038/srep32371)
Supplement: Supplementary Information [file srep32371-s1.pdf]

**<u>Supplement: procedures, tables, figure and movie legends as well as figures</u>**

# The BR domain of PsrP interacts with extracellular DNA to promote bacterial aggregation; structural insights into pneumococcal biofilm formation

Tim Schulte[*], Cecilia Mikaelsson[*], Audrey Beaussart[Δ,∂], Alexey Kikhney[‡], Maya Deshmukh[*], Sebastian Wolniak[*], Anuj Pathak[†], Christine Ebel[§], Jonas Löfling[†], Federico Fogolari[f], Birgitta Henriques-Normark[†], Yves F. Dufrêne[Δ], Dmitri Svergun[‡], Per-Åke Nygren[#] and Adnane Achour[*]

[*] Science for Life Laboratory, Department of Medicine Solna, Karolinska Institute, and Department of Infectious Diseases, Karolinska University Hospital, Solna, SE-17176 Stockholm, Sweden

[Δ] Université catholique de Louvain, Institute of Life Sciences, Croix du Sud, 4-5, bte L7.07.06, B-1348 Louvain-la-Neuve, Belgium

[∂] current affiliation: CNRS, LIEC (Laboratoire Interdisciplinaire des Environnements Continentaux), UMR 7360, Vandoeuvre-les-Nancy, F-54501, France

[‡] European Molecular Biology Laboratory (EMBL), Hamburg Outstation, Notkestrasse 85, 22603 Hamburg, Germany

[†] Department of Microbiology, Tumor and Cell Biology (MTC), Karolinska Institute; Clinical Microbiology, Karolinska University Hospital Solna, Stockholm, Sweden

[§] Institut de Biologie Structurale (IBS), Univ. Grenoble Alpes, CEA, CNRS, 38044 Grenoble, France

[#] Division of Protein Technology, School of Biotechnology, KTH–Royal, Institute of Technology, Sweden

[f] Dipartimento di Scienze Mediche e Biologiche, Universita' di Udine, Piazzale Kolbe 4, 33100 Udine – Italy

## Supplemental experimental procedures

### CD spectroscopy

Protein samples were diluted to give a final absorption of 0.9 and 1.1 for $BR^*_{120-395}$ and $BR_{187-378}$ in 10mM $KPO_4$, 100mM $(NH_4)_2SO_4$, pH 7.2 in a 1 cm pathlength cuvette. CD spectra of the samples 0.1 mm cuvettes (Hellma) were recorded on a JASCO J-820 CD-Spectropolarimeter (Jasco) at 25 °C. The final spectra were accumulated from eight measurements (bandwidth 1 nm, step 0.2 nm, 2s response time) and a Savitzky-Golay filter was applied two times over 25 data points. The spectrum of the buffer was subtracted and data were converted to mean residue ellipticity according to formulas given in (4). Secondary structure contributions were calculated using the CDSSTR algorithm with reference protein set 4 and 7 as implemented in Dichroweb (5). Almost identical secondary structure contributions for the spectra of $BR^*_{120-395}$ and $BR_{187-378}$ were derived when protein reference set 4 was used for spectral deconvolution. Usage of reference set 7 resulted in significant differences and was considered as more reasonable given the clear differences in the CD spectra.

### SAXS Data analysis.

The forward scattering I(0), the radius of gyration $R_g$ along with the pair distribution function of the particle p(r) and the maximum dimension $D_{max}$ were derived using the automated SAXS data analysis pipeline (2). The p(r) functions were refined manually with the program GNOM (6). The molecular weights (MW) were evaluated by comparison of the forward scattering with that from a bovine serum albumin (MW = 66 kDa) reference solution. Experimental data plotted as $s^2I(s)$ versus s (Kratky plot)

allows estimating the degree of flexibility of proteins. If the intensity is normalized by I(0) and s is multiplied by $R_g$ the Kratky plot becomes dimensionless and can be used to compare the foldness of proteins independently of the protein size (7). The theoretical curves for the globular protein and the random chain were calculated using the Guinier law $I(s)/I(0) = \exp[-(sR_g)^2/3]$ and the Debye law $I(s)/I(0) = 2 (x - 1 + \exp [-x ]) / x^2$ where $x = (sR_g)^2$. The excluded volumes of the hydrated proteins were computed with the program AUTOPOROD (8). For globular proteins the hydrated volumes in $Å^3$ are about 1.6 times the MW in Da.

**Force spectroscopy using AFM**

*Preparation of functionalized surfaces.* $BR_{187-385}$ and $BR^*_{120-395}$ were covalently immobilized, in a random orientation, onto self-assembled monolayers (SAMs) of carboxyl-terminated alkanethiols. Silicon wafers (Siltronix, France) were coated by thermal evaporation with a 5 nm thick Cr layer followed by a 30 nm thick Au layer, yielding gold surfaces with ~ 1 nm roughness. Gold surfaces were immersed overnight in ethanol solutions containing 1 mM of $HS(CH_2)_{15}COOH$ (16-mercaptohexadecanoic acid) and $HS(CH_2)_{11}OH$ (11-mercapto-1-undecanol) (0.1:0.9) (Sigma), rinsed with ethanol, dried with $N_2$, immersed for 30 min in a solution containing 10 mg/ml N-hydroxysuccinimide (NHS) (Sigma) and 25 mg/ml 1-ethyl-3-(3-dimethylaminopropyl)-carbodiimide (EDC) (Sigma), and rinsed with MilliQ water. Surfaces were then covered with a 100 µL droplet of a buffer solution (20 mM Hepes, 150 mM NaCl, pH 7.5) containing proteins (70 µM), allow to stand for 2 hours, rinsed 3 times and stored in buffer solution.

*AFM tips functionalization.* AFM tips were functionalized with $BR_{187\text{-}385}$ and $BR^*_{120\text{-}395}$ residues using ~6 nm long PEG-benzaldehyde linkers as described by Ebner and coworkers (9). Oxide-sharpened microfabricated $Si_3N_4$ cantilevers (MSCT, Bruker, nominal spring constant of 0.01 N/m) were placed in an UV-ozone-cleaner for 15 min, immersed in piranha solution for 20 minutes, and abundantly rinsed with deionized water. Cantilevers were then washed with chloroform and ethanol, placed in an UV-ozone-cleaner for 15 min, rinsed with ethanol, dried with $N_2$ and immersed overnight in an ethanolamine solution (3.3 g of ethanolamine dissolved in 6 mL of DMSO), washed three times with DMSO, twice with ethanol, and dried. The ethanolamine-coated cantilevers were immersed for 2 h in a solution prepared by mixing 1 mg Acetal-PEG-NHS dissolved in 0.5 mL chloroform with 10 µL triethylamine, then washed with chloroform and dried with $N_2$. Cantilevers were further immersed for 10 min in a 1% citric acid solution, washed in MilliQ water, and then covered with a 100 µL droplet of a buffer solution (20 mM Hepes, 150 mM NaCl, pH 7.5) containing residues (70 µM) to which 2 µL of a 1 M $NaCNBH_3$ solution were added. After 50 min, cantilevers were incubated for 10 min with 5 µL of a 1 M ethanolamine solution in order to passivate unreacted aldehyde groups, and then washed with and stored in buffer.

*Single-molecule force spectroscopy.* SMFS measurements were performed using a Multimode VIII AFM (Bruker, Santa Barbara, CA), at room temperature in buffer solution (20 mM Hepes, 150 mM NaCl, pH 7.5). Functionalized surfaces were attached to a steel sample puck using a small piece of double-face adhesive tape, and the mounted sample was transferred into the AFM liquid cell while avoid dewetting. The cantilevers

spring constants were measured by the thermal noise method (Picoforce, Bruker). Adhesion maps were obtained by recording 32 x 32 force distance curves on areas of given size (5 µm x 5 µm) and calculating the adhesion force for each force curve. All curves were recorded using a maximum applied force of 250 pN, a contact time of 500 ms, and constant approach and retraction speeds of 1000 nm $s^{-1}$, unless otherwise stated.

**Crystallization, data collection and crystal structure determination**

The $BR_{187\text{-}378}$ dimer was concentrated to 20 mg/mL in 20 mM Na-Citrate, 500 mM NaCl, 10% (V/V) glycerol, pH 5.5. Well-diffracting crystals were obtained in 100 mM Trisodiumcitrate dihydrate pH 5.6; 34-44% 2-methyl-4-pentanediole using the sitting drop vapor-diffusion method. Crystals were flash-frozen in liquid nitrogen. X-ray diffraction data was collected at beam line ID23-1 at the synchrotron radiation facility at the ESRF (Grenoble, France). Crystals diffracted to 2.1 Å resolution and were processed using the XDS program package (10) (Table 2). A molecular replacement search performed using Phaser (11) revealed three molecules of the $BR_{187\text{-}385}$ monomer (PDB: 3ZGH) within the asymmetric unit. Initial rigid body and restrained refinement rounds were performed in CCP4 refmac, and Coot was used for manual model rebuilding (12, 13). The mFo-DFc electron density difference map clearly indicated that residues Q312-G315 had to be re-built such that residues L202-G315 of chain A were linked to residues Y316-S377 of the symmetry mate [X, -Y, -Z + (1 0 0) & {-1 0 0}), symmetry operation in Coot], and *vice versa*. The same procedure was applied to swap the corresponding regions between chains B and C. The model was thereafter refined using Phenix (14) with individual isotropic ADP factors, TLS refinement and NCS torsion restraints. The

final model was refined to R and $R_{free}$ values of 21.1 and 24.4, respectively, and comprised residues L202-S377, residues N203-S377 and residues N203-S376 for chains A, B and C, respectively. The electron density for the structural model of chain A was of higher quality compared to the density for both chains B and C, which was corroborated by higher values for real-space correlation coefficients. This difference in the quality of the electron density map was also true for the crucial loop region around residue S314.

**Structural analysis**

The simulated annealing composite electron density map displayed in Figure S2 was calculated using autobuild in Phenix (14). Secondary structure analyses were performed using PDBsum (15) and 2Struc (16).

**Molecular dynamics simulations**

An arrangement similar to the complex obtained from the 'ParaDock' webserver was obtained using the program 'vina' (17), rigidly docking a short DNA fragment of 10 base-pairs onto the $BR_{187-385}$ dimer, with few bumps similar to the 'ParaDock' docking complexes.

The best model from 'ParaDock' docking was subjected to molecular dynamics simulations in order to check whether, on a timescale of tens of nanoseconds, the complex would be stable or spontaneously dissociate. The structure of the complex was prepared using the psfgen utility of the software package NAMD (18). The forcefield CHARMM22 (19) with the CMAP correction for backbone torsion angles (20) was used. The complex was solvated using the software VMD (21). 44 sodium ions were added

using the same software in order to neutralize the system. The system was first energy minimized keeping protein atoms fixed by 300 minimization steps and the solvent was simulated for 20 ps in order to let it soak the solute molecule. Then the whole system was energy minimized by 300 minimization steps and molecular dynamics simulation was run at constant temperature and pressure.

The temperature (310 K) control was effected by Langevin dynamics and the pressure (1.012E05 Pa) was controlled using a Langevin piston. Electrostatics was treated by a simple cutoff (14 Å) scheme. The system was simulated for 50 ns. Due to forcefield inaccuracy and simple treatment of electrostatics the molecule of DNA tends to distort at the end of simulation. We analysed snapshots taken at 100 ps intervals along the simulation between 10 and 40 ns.

**Surface display of BR on the surface of *Staphylococcus carnosus***

*Cloning*. The PCR-amplified PsrP constructs and the gene-synthesized (Eurofins Genomics, Germany) DUF1542 repeat domain of SasC (Uniprot ID: C7BUR8) were cloned into the staphylococcal display vector 'pHis3C' using a sequence and ligation independent cloning (SLIC) method (22, 23). The vector comprises two origins of replication, oriE and oriS, as well as ampicillin and chloramphenicol resistance marker genes for vector construction in *Escherichia coli* XL1 blue (Agilent Technologies, USA) and protein surface display using *Staphylococcus carnosus* TM300 (23), respectively. Coding sequences of the protein-display constructs were confirmed by DNA sequencing and are listed in the supplemental information.

*Cellular aggregation assay.* Transformed *S. carnosus* cells were cultured in TB medium with chloramphenicol (10ug/mL) overnight at 37°C at 160 rpm. Cells were adjusted to $OD_{600nm}$ ~2 and split into two separate samples, one of which was treated with DNaseI (A3778, Applichem, Germany) at a final concentration of 0.6 μM in the presence of 5 mM $MgCl_2$. Cells were incubated for another hour at 37°C at 160 rpm before harvesting by centrifugation (3750g, 4°C, 10 minutes) and gently re-suspended in PBS. Re-suspended cells were adjusted to $OD_{600nm}$ ~0.25 in PBS. After a further one-to-eight dilution, a volume of 150 μL was filled into wells of a 96-μ-well plate (Ibidi, Germanu) and covered with 25 μL of silicone oil AR 200 (Sigma Aldrich, USA). After 3h equilibration at RT, images were taken using the ZOE Fluorescent Cell Imager (Biorad, USA) at 20x magnification. Images were analyzed using the software cell profiler (www.cellprofiler.org), the particle parameter values were imported into the R software package for statistical analysis and visualization using density histogram and q-q plots (24, 25).

*Polyclonal rabbit-antisera against $BR_{187-385}$ and $BR_{143-156}$.* Polyclonal antibodies against the TEV-cleaved purified $BR_{187-385}$ monomer and a synthesized $BR_{143-156}$ (RKKPASDYVASVTN) peptide were raised in rabbits and the obtained sera yielded titers of about 75000 and 6000, respectively (Innovagen, Sweden). For fluorescent staining, anti- $BR_{187-385}$ antibodies were affinity-purified using a $BR_{187-385}$-immobilized column according to the manufacturers instructions (HiTrap NHS HP, GE healthcare). The harvested PBS-resuspended bacteria were diluted to a concentration of approximately $1x10^8$ bacteria/ml, incubated at 37°C for 30 min with anti BR antibody at a

dilution of 1:1000. After three wash steps the surface-displayed proteins were detected using fluorescein isothiocyanate (FITC)-labeled goat anti-rabbit IgG (life technologies) secondary antibody used at a dilution of 1:200. Each incubation step was followed by three washes in PBS (centrifugation at 1500 *g* for 5 min). Bacteria were fixed in 4% paraformaldehyde, washed, and visualized using a fluorescence microscope (Leica Leitz DMRBE).

*Dotblot assays.* 5 mL overnight cultures of *S. carnosus* cells were harvested by centrifugation (4000 g, 4°C, 10 min), washed and resuspended in cleavage buffer (50mM Tris-HCl, 150mM NaCl, 0.1% Tween-20, 5 mM $MgCl_2$ pH 7.0) to a final volume of 500 μL. DTT was added to a final concentration of 5 mM, together with 4U of PreScission protease (GE Healthcare Bio-sciences AB, Uppsala, Sweden), and samples were incubated with gentle mixing at 4°C for 24 h. The cells were harvested by centrifugation, and the supernatants were concentrated using Vivaspin turbo protein concentration devices with a MW-cutoff of 3 kDa (Vivapsin Turbo 3 kDa, Sartorius) to a volume of 50 μL. Samples with a volume of two microliter were applied on nitrocellulose filter membranes and blocked 1h at RT using Pierce Protein-free blocking buffer (Thermo Scientific). Washing steps were performed using PBS supplemented with 0.05% Tween-20 (PBS-T), and all incubations and washing steps were performed with gentle shaking. The membranes were incubated overnight at 4°C using polyclonal rabbit-antisera against $BR_{187\text{-}385}$, $BR_{143\text{-}156}$ as well as HRP-coupled anti-His antibodies (ab1187; Abcam, UK), at dilutions of 1:5000 and 1:1000 as well as 1:2500 in PBS-T, respectively. The anti-BR antibody stained samples were incubated using HRP-coupled monoclonal anti-rabbit

IgGγ (A1949, Sigma Aldrich) for 4h at RT at a dilution of 1:4000. Membranes were developed using Pierce ECL western blotting substrate (Thermo Scientific) and the chemiluminescent signal was detected using a Chemidoc XRS+ system (Biorad).

**Translated coding sequences of His-$BR_{187-385}$, His-$BR_{187-378}$, His- $BR_{120-395}$, His-$BR^{*}_{120-395}$**

> His-$BR_{187-385}$

HHHHHHSGNTIVNGAPAINASLNIAKSETKVYTGEGVDSVYRVPIYYKLKVTND
GSKLTFTYTVTYVNPKTNDLGNISSMRPGYSIYNSGTSTQTMLTLGSDLGKPSGV
KNYITDKNGRQVLSYNTSTMTTQGSGYTWGNGAQMNGFFAKKGYGLTSSWTV
PITGTDTSFTFTPYAARTDRIGINYFNGGGKVVESSTTSQSLSQ

> His-$BR_{187-378}$

HHHHHHSGNTIVNGAPAINASLNIAKSETKVYTGEGVDSVYRVPIYYKLKVTND
GSKLTFTYTVTYVNPKTNDLGNISSMRPGYSIYNSGTSTQTMLTLGSDLGKPSGV
KNYITDKNGRQVLSYNTSTMTTQGSGYTWGNGAQMNGFFAKKGYGLTSSWTV
PITGTDTSFTFTPYAARTDRIGINYFNGGGKVVESST

> His- $BR_{120-395}$

HHHHHHSSTVVGSQTAAATEATAKKVEEDRKKPASDYVASVTNVNLQSYAKRRKRSVDS
IEQLLASIKNAAVFSGNTIVNGAPAINASLNIAKSETKVYTGEGVDSVYRVPIYYKLKVTND
GSKLTFTYTVTYVNPKTNDLGNISSMRPGYSIYNSGTSTQTMLTLGSDLGKPSGVKNYITD
KNGRQVLSYNTSTMTTQGSGYTWGNGAQMNGFFAKKGYGLTSSWTVPITGTDTSFTFTP
YAARTDRIGINYFNGGGKVVESSTTSQSLSQSKSLSVSASQ

> His- BR*$_{120-395}$

HHHHHHSSTVVGSQTAAATEATAKKVEEDRKKPASDYVASVTNVNLQSYAKSR

KSSVDSIEQLLASIKNAAVFSGNTIVNGAPAINASLNIAKSETKVYTGEGVDSVYR

VPIYYKLKVTNDGSKLTFTYTVTYVNPKTNDLGNISSMRPGYSIYNSGTSTQTML

TLGSDLGKPSGVKNYITDKNGRQVLSYNTSTMTTQGSGYTWGNGAQMNGFFAK

KGYGLTSSWTVPITGTDTSFTFTPYAARTDRIGINYFNGGGKVVESSTTSQSLSQS

KSLSVSASQ

**PCR-amplified and gel-extracted 276 bp long DNA molecule**

> DNA$_{276bp}$

AGCGCGTGGAGCCATCCGCAGTTTGAAAAAGAAAACCTGTACTTCCAGGGTC

AGATCTCCAACTTGCAGCAGTCCATCAGTGATGCAGAGCAGCGTGGCGAGAA

TGCCCTCAAGGATGCCAAGAACAAGCTGAATGACCTGGAGGATGCCCTGCAG

CAGGCCAAGGAAGACCTGGCCCGCCTGCTGCGCGACTACCAGGAGCTGATGA

ACACCAAGCTGGCCCTGGATCTGGAGATTGCCACCTACAGGACCCTCCTGGA

GGGATAATTAACCTAG

**Translated coding sequences of the surface display vector-encoded --S-PP-[insert]-His$_6$-3C-DUF-XM-- and the inserts BR$_{187-378}$, BR*$_{120-163}$, and BR*$_{120-378}$**

>> PP- BR*$_{120-378}$ - 3C-His$_6$ linker - DUF1542

>PP

NDQTTQTTTPLEVAQTSQQETHTHQTPVTSLHTATPEHVDDSKEATPLPEKAESP

KTEVTVQPSSHTQEVPALHKKTQQQPAYKDKTVPESTIASKSVESNKATENEMSP

VEHHASNVEKREDRLETNETTPPSVDREFSHKIINNTHVNPKTDGQTNVNVDTKT

IDTVSPKDDRIDTAQPKQVEAPKENTTAQNKFTSQASDKKPT

>BR*$_{120\text{-}378}$ (**BR*$_{185\text{-}378}$**)

SSTVVGSQTAAATEATAKKVEEDRKKPASDYVASVTNVNLQSYAKSRKSSVDSI

EQLLASIKNAAVF**SGNTIVNGAPAINASLNIAKSETKVYTGEGVDSVYRVPIYY**

**KLKVTNDGSKLTFTYTVTYVNPKTNDLGNISSMRPGYSIYNSGTSTQTMLTL**

**GSDLGKPSGVKNYITDKNGRQVLSYNTSTMTTQGSGYTWGNGAQMNGFFA**

**KKGYGLTSSWTVPITGTDTSFTFTPYAARTDRIGINYFNGGGKVVESST**

> 3C-His$_6$ linker

ASTHHHHHHEALFQGPASL

> DUF1542

QHIAEINANPDATQEERQAAIDKVNAAVTAANTNILNANTNADVEQVKTNAIQG

IQAITPATKVKTDAKNAIDKSAETQHNTIFNNNDATLEEQQAAQQLLDQAVATA

KQNINAADTNQEVAQAKDQGMQNIVVIQPATQVKTDARNTVNEKAREAITNIN

ATPGATREEKQEAIDRVNALKNRALTDIGVTSTTAMVNSIRDDAVNQIGAVQPH

VTKKQTATGVLNDLATAKKQEINQNTNATTEEKQMALNQVDQDLATAINNINQ

ADTNTEVDQAQQLGAQAINAIQPNIVKKPAALAQINQHYNAKLAEINATPDATD

DEKNAAINTLNQDRQQAIESVKQANTNNEVDQAATTAENNIDAVQVDVVKKQA

ARDKITAEVAKRIEAVKQTPNATDEEKQAAVNQINQLKDQAFNQINQNQTNDQV

DTTTNQALNAIDNVEAEVVIKPKAIADIEKAVKEKQQQIDNSLDSTDNEKEVASQ

ALAKEKEKALAAIDQAQTNSQVNQAATNGVSAIKIIQPETKVKPAAREKINQKA

NELRAKINQDKEATAEERQVALDKINEFVNQAMTDITNNRTNQQVDDTTSQALD

SIALVAPEHIARAAARDAVKQQ

**References**


1. Pernot P, Round A, Barrett R, De Maria Antolinos A, Gobbo A, Gordon E, Huet J, Kieffer J, Lentini M, Mattenet M, Morawe C, Mueller-Dieckmann C, Ohlsson S, Schmid W, Surr J, Theveneau P, Zerrad L, McSweeney S. 2013. Upgraded ESRF BM29 beamline for SAXS on macromolecules in solution. J Synchrotron Radiat 20:660–664.

2. Franke D, Kikhney AG, Svergun DI. 2012. Automated acquisition and analysis of small angle X-ray scattering data. Nucl Instrum Methods Phys Res Sect Accel Spectrometers Detect Assoc Equip 689:52–59.

3. Bernadó P, Mylonas E, Petoukhov MV, Blackledge M, Svergun DI. 2007. Structural characterization of flexible proteins using small-angle X-ray scattering. J Am Chem Soc 129:5656–5664.

4. Greenfield NJ. 2007. Using circular dichroism spectra to estimate protein secondary structure. Nat Protoc 1:2876–2890.

5. Whitmore L, Wallace BA. 2004. DICHROWEB, an online server for protein secondary structure analyses from circular dichroism spectroscopic data. Nucleic Acids Res 32:W668-673.

6. Svergun DI. 1992. Determination of the regularization parameter in indirect-transform methods using perceptual criteria. J Appl Crystallogr 25:495–503.

7. Durand D, Vivès C, Cannella D, Pérez J, Pebay-Peyroula E, Vachette P, Fieschi F. 2010. NADPH oxidase activator p67phox behaves in solution as a multidomain protein with semi-flexible linkers. J Struct Biol 169:45–53.

8. Petoukhov MV, Franke D, Shkumatov AV, Tria G, Kikhney AG, Gajda M, Gorba C, Mertens HDT, Konarev PV, Svergun DI. 2012. New developments in the ATSAS program package for small-angle scattering data analysis. J Appl Crystallogr 45:342–350.

9. Ebner A, Wildling L, Kamruzzahan ASM, Rankl C, Wruss J, Hahn CD, Hölzl M, Zhu R, Kienberger F, Blaas D, Hinterdorfer P, Gruber HJ. 2007. A New, Simple Method for Linking of Antibodies to Atomic Force Microscopy Tips. Bioconjug Chem 18:1176–1184.

10. Kabsch W. 1993. Automatic processing of rotation diffraction data from crystals of initially unknown symmetry and cell constants. J Appl Crystallogr 26:795—800.

11. McCoy AJ. 2007. Solving structures of protein complexes by molecular replacement with Phaser. Acta Crystallogr D Biol Crystallogr 63:32–41.

12. Emsley P, Cowtan K. 2004. Coot: model-building tools for molecular graphics. Acta Crystallogr D Biol Crystallogr 60:2126–2132.

13. CCP4. 1994. The CCP4 suite: programs for protein crystallography. Acta Crystallogr D Biol Crystallogr 50:760–763.

14. Adams PD, Grosse-Kunstleve RW, Hung LW, Ioerger TR, McCoy AJ, Moriarty NW, Read RJ, Sacchettini JC, Sauter NK, Terwilliger TC. 2002. PHENIX: building new software for automated crystallographic structure determination. Acta Crystallogr D Biol Crystallogr 58:1948–1954.

15. Laskowski RA. 2009. PDBsum new things. Nucleic Acids Res 37:D355-359.

16. Klose DP, Wallace BA, Janes RW. 2010. 2Struc: the secondary structure server. Bioinformatics 26:2624–2625.

17. Trott O, Olson AJ. 2010. AutoDock Vina: improving the speed and accuracy of docking with a new scoring function, efficient optimization, and multithreading. J Comput Chem 31:455–461.

18. Kalé L, Skeel R, Bhandarkar M, Brunner R, Gursoy A, Krawetz N, Phillips J, Shinozaki A, Varadarajan K, Schulten K. 1999. NAMD2: Greater Scalability for Parallel Molecular Dynamics. J Comput Phys 151:283–312.

19. MacKerell AD, Bashford D, Bellott M, Dunbrack RL, Evanseck JD, Field MJ, Fischer S, Gao J, Guo H, Ha S, Joseph-McCarthy D, Kuchnir L, Kuczera K, Lau FT, Mattos C, Michnick S, Ngo T, Nguyen DT, Prodhom B, Reiher WE, Roux B, Schlenkrich M, Smith JC, Stote R, Straub J, Watanabe M, Wiórkiewicz-Kuczera J, Yin D, Karplus M. 1998. All-atom empirical potential for molecular modeling and dynamics studies of proteins. J Phys Chem B 102:3586–3616.

20. Mackerell AD, Feig M, Brooks CL. 2004. Extending the treatment of backbone energetics in protein force fields: limitations of gas-phase quantum mechanics in reproducing protein conformational distributions in molecular dynamics simulations. J Comput Chem 25:1400–1415.

21. Humphrey W, Dalke A, Schulten K. 1996. VMD: Visual molecular dynamics. J Mol Graph 14:33–38.

22. Li MZ, Elledge SJ. 2007. Harnessing homologous recombination in vitro to generate recombinant DNA via SLIC. Nat Methods 4:251–256.

23. Kronqvist N, Löfblom J, Severa D, Ståhl S, Wernérus H. 2008. Simplified characterization through site-specific protease-mediated release of affinity proteins selected by staphylococcal display. FEMS Microbiol Lett 278:128–136.

24. Jones TR, Kang IH, Wheeler DB, Lindquist RA, Papallo A, Sabatini DM, Golland P, Carpenter AE. 2008. CellProfiler Analyst: data exploration and analysis software for complex image-based screens. BMC Bioinformatics 9:482.

25. R Core Team. R: A language and environment for statistical computing. R Foundation for Statistical Computing, Vienna, Austria.

**Table S1. SAXS and AUC data collection and analysis parameters**

| | $BR_{187\text{-}385}$ monomer | $BR_{120\text{-}385}$ monomer |
|---|---|---|
| **SAXS data collection parameters** | | |
| **instrument** | beamline BM29 at ESRF, Grenoble (1) | |
| **exposure time per frame (s)** | 1 x 10 frames per sample | |
| **concentration range (mg/ml)** | 0.3 – 3.0 | |
| **temperature (K)** | 293 | |
| **SAXS structural parameters** | | |
| **$R_g$ (Å) (from Guinier approximation)** | 20 ± 1 | 29 ± 2 |
| **$R_g$ (Å) (from p(r))** | 21 ± 2 | 31 ± 3 |
| **$D_{max}$ (Å)** | 78 ± 8 | 125 ± 12 |
| **molecular mass determination** | | |
| **MW (kDa) from I(0)** | 17 ± 2 | 20 ± 2 |
| **MW (kDa) from Porod volume** | 24 ± 2 | 26 ± 3 |
| **MW (kDa) from sequence** | 22.1 | 30.3 |
| **software employed** | | |
| **primary data reduction and processing** | automated SAXS data analysis pipeline (2) | |
| **flexibility analysis** | EOM (3) | |
| **AUC analysis** | | |
| **sedimentation coefficient (S)** | 1.84 ± 0.03 | 1.82 ± 0.03 |
| **$s_{20w}$ , corrected to 20°C in water** | 2.11 ± 0.03 | 2.10 ± 0.03 |
| **MW from non-interacting species analysis (kDa)** | 21.1 | 27.3 |
| **$f/f_{min}$** | 1.4 | 1.7 |
| **HYDROPRO** | | |
| **$R_g$-value of model used for calculations (Å)** | 21 | 30 |
| **sedimentation coefficient (S)** | 2.10 | 2.19 |

**Table S2. Crystal structure data collection and refinement statistics of the domain-swapped $BR_{187-378}$ dimer**

| | |
|---|---|
| **data collection** | |
| **space group** | $C222_1$ |
| **unit cell parameters (a, b, c in Å; α, β, γ in °)** | a = 109.8; b = 162.4; c = 102.1; α = β = γ = 90 |
| **X-Ray source** | ESRF ID23-1 |
| **detector** | Pilatus 6M |
| **temperature (K)** | 100 |
| **resolution limits (Å)** † | 48.4- 2.1 (2.18-2.1) |
| **wavelength (Å)** | 0.95376 |
| **no. of observations** | 355483 (35922) |
| **no. of unique reflections** | 53483 (5636) |
| **redundancy** | 6.6 (6.4) |
| **completeness (%)** | 100 (100) |
| **I / σ** | 16.1 (2.0) |
| **$R_{sym}$ (%)** | 5.9 (86.4) |
| **$R_{meas}$ (%)** | 6.4 (94.1) |
| **$CC_{1/2}$** | 100 (72.2) |

| | |
|---|---|
| **structure refinement** | |
| **PDB Entry** | 5JUI |
| **molecules in ASU** | 3 |
| **mask estimated solvent (%)** | 67.4 |
| **$R_{work}$ (%)** | 21.1 |
| **$R_{free}$ (%)** | 24.4 |
| **no. of residues** | |
| **protein** | 525 |
| **water** | 207 |
| **small molecule** | 1 |
| **other** | 1 |
| **no. of atoms** | 4236 |
| **mean isotropic B-value (Å²)** | 65.3 |
| **Wilson B-factor (Å²)** | 49.1 |
| **Rmsd from ideal bond lengths (Å)** | 0.003 |
| **Rmsd from ideal bond angle (°)** | 0.848 |
| **Molprobity** | |
| **Ramachandran** | |
| **outliers (%)** | 0 |
| **allowed (%)** | 3 |
| **favored (%)** | 97 |
| **all atom clash score (percentile)** | 5.91 ($97^{th}$) |
| **Molprobity score (percentile)** | 1.51 ($98^{th}$) |

**Figure S1. Analytical ultracentrifugation analysis confirms stable $BR_{187\text{-}385}$ monomer and dimer as well as $BR^*_{120\text{-}395}$ monomer populations**

Sedimentation velocity analytical ultracentrifugation experiments of (A) $BR_{187\text{-}385}$ monomer and dimer as well as (B) $BR^*_{120\text{-}395}$ monomer fractions were analysed in terms of distributions of sedimentation coefficients $c(s)$ revealing sedimentation coefficients of 1.84 ± 0.03 S and 2.71 +/- 0.06 as well as 1.82 +/- 0.03 S, respectively.

**Figure S2. The crystal structure of the irreversibly associated $BR_{187\text{-}378}$ dimer reveals a three-dimensional domain swap mechanism**

(A) Each subdomain within the $BR_{187\text{-}378}$ dimer is created by two separate chains, but shares the same MSCRAMM-related DEv-IgG fold previously described for the $BR_{187\text{-}385}$ monomer (displayed in B). The altered conformation of the "hinge" loop in the dimer creates a new secondary interface with strands D2 and D2* forming a two-stranded antiparallel β-sheet.

(B) The MSCRAMM-related DEv-IgG fold of the $BR_{187\text{-}385}$ monomer was reproduced from Figure 3 presented in (Schulte T *et al.* Open Biol **4**:130090). The hinge loop that has a different conformation in the dimer is highlighted.

(C) The structure of the $BR_{187\text{-}385}$ monomer (black) was superimposed on each subdomain of the $BR_{187\text{-}378}$ dimer (green) with a root mean square deviation (rmsd) of 0.6 Å. The two chains of the presented $BR_{187\text{-}378}$ dimer are created from chain A and a symmetry neighbor. The new path of the "hinge" loop comprising residues $Gly_{313}$-$Ser_{314}$-$Gly_{315}$ is evident from the 2mFo-DFc composite omit electron density map presented at a σ-level of 1.5. The residues of the dimer and the superimposed monomer are displayed in green and black, respectively.

**Figure S3. BR*$_{120\text{-}186}$ forms a non-globular, flexible structure as revealed in SAXS-derived distance distribution and Kratky plots as well as from CD analysis.**

(A) The radii of gyration ($R_g$) derived from the shown Guinier plots were 20 ± 1 Å and 29 ± 2 Å for BR$_{187\text{-}385}$ and BR$_{120\text{-}395}$.

(B) The distance distribution functions p(r) of BR$_{187\text{-}385}$ and BR*$_{120\text{-}395}$ gave $R_g$ values of 20 ± 2 Å and 31 ± 3 Å, and $D_{max}$ values of 78 ± 8 Å and 125 ± 12 Å, respectively.

(C) Dimensionless Kratky plots of the scattering data revealed that the BR$_{187\text{-}385}$ profile matched more closely the curve expected for globular proteins while the BR*$_{120\text{-}395}$ profile was shifted towards the curve expected for a disordered, random chain. A fully folded monodomain globular protein typically features a peak at 1.1 at $sR_g = \sqrt{3}$ (dotted line).

(D) The CD spectra of BR$_{187\text{-}385}$ and BR*$_{120\text{-}395}$ are represented as mean residue ellipticities. The broad negative peak with a minimum at 212 nm is characteristic for β-strands. The atypical positive ellipticity peaks at 230 nm possibly originate from aromatic chromophores (Khan MY *et al.* 1989. J Biol Chem 264:2139–2142). The listed secondary structure contributions were obtained by spectral deconvolution. The secondary structure contributions derived from the crystal structure of BR$_{187\text{-}385}$ are 2% α-helices, 43% β-strand, 17% turns and 38% disordered (Schulte T *et al.* Open Biol **4**:130090). The (BR*$_{120\text{-}395}$ - BR$_{187\text{-}385}$) difference spectrum highlights the negative ellipticity peak at 200 nm characteristic for disordered structures.

**Figure S4. Intermolecular β-sheet interfaces are also observed for the Seleno-methionine derivative of $BR_{187\text{-}385}$ and the domain-swapped dimers of $BR_{187\text{-}378}$.**

(A) A saddle-like dimer with ISA of 440 $Å^2$ similar to the one of native $BR_{187\text{-}378}$ is observed in the $P4_32_12$ crystal structure of the Selenomethionine derivative (PDB: 3ZGI). However, the b-strands are paired with a frame shift of two residues, e.g. Glu-208 pairs with Lys-210 in the 3ZGH structure, but with Glu-208 in 3ZGI. The two other chains B and C in the 3ZGI crystal structure form the same b-strand pairs as shown for 3ZGH (Figure 3), revealing that both b-strand pairings are observed in the same crystal.

(B, C) Intermolecular b-sheets were also observed between symmetry-related subdomains of two domain-swapped dimers. The subdomains (B) AI/AII$^{*1}$ and AI$^{*2}$/AII$^{*3}$ as well as (C) BII/CI and BI$^{*4}$/CII$^{*4}$ form intermolecular b-sheets with the same residue pairings as the pseudo-dimers shown in Figure 3, with eight and eleven hydrogen bonds between the backbones of the two A1-strands, respectively. The total ISA between the two subdomains are 640 $Å^2$ and 510 $Å^2$. Symmetry-related molecules are labeled with an asterix and a unique number (e.g. $*^1$). In the figures only the b-sheet forming subdomains are shown. In the upper panel, the position of the second subdomain of each domain-swapped dimer is indicated with dashed lines.

**Figure S5. Force spectroscopy and dynamics of homophilic $BR_{187-385}$ and $BR^{*}_{120-395}$ interactions**

(A) AFM tips and substrates were functionalized with randomly oriented $BR_{187-385}$ and $BR^{*}_{120-395}$ to measure their homophilic interaction forces.

(B) AFM height image (z=10 nm) recorded with a silicon nitride tip documenting the presence of a smooth homogeneous layer of BR proteins.

(C) Force histograms with representative force profiles revealed mean adhesion forces of 70 ± 18 pN and 68 ± 14 pN for $BR_{187-378}$ and $BR^{*}_{120-395}$, respectively. Histograms were obtained from 2048 force curves from 2 independent experiments. All curves were obtained with a maximum applied force of 250 pN, a contact time of 500 ms, and an approach and retraction speed of 1,000 nm/s.

(D) The adhesion forces of both $BR_{187-378}$ and $BR^{*}_{120-395}$ increased linearly with the logarithm of the loading rate, as expected for specific bimolecular bonds. The dependence of the adhesion force on the loading rate applied during retraction was measured using a contact time of 100 ms, a maximum applied force of 250 pN and an approach speed of 1,000 nm/s (each point is extracted from 1024 force measurements at a given retraction speed).

**Figure S6. Addition of eDNA to *S. carnosus* displaying $BR_{187-385}$ and $BR^*_{120-378}$ grown in M9 minimal medium significantly enhanced bacterial aggregation**

(A) Phase-like contrast microscopy images revealed the formation of cellular aggregates of *S. carnosus* displaying $BR_{187-378}$ and $BR^*_{120-378}$, when bacteria were grown in M9 minimal medium supplemented with eDNA. Cells displaying the DUF domain alone were not affected by the addition of eDNA.

(B) Absence of DNA in the medium significantly reduced the bacterial cell aggregates of Scar- $BR_{187-378}$. Scar-DUF cells appeared similar as the ones in the presence of eDNA.

(G) Q-q plots reveal that Scar-$BR_{187-378}$ and Scar-$BR_{120-378}$ were significantly more aggregated when bacteria were grown in M9 minimal medium with the addition of eDNA (red and blue, respectively), compared to growth in the absence of eDNA (light and dark green, respectively). Scar-DUF was not affected by the addition of DNA, and had a very similar particle size distribution as shown in Figure 5G. For comparison, qq-plots of Scar-$BR_{120-378}$ grown in TB without and with DNase treatment (as semi-transparent blue and green lines, respectively, taken from Figure 5G) are included. The q-q plot of Scar-DUF grown in TB is also included (semi-transparent black line), but identical to the q-q plots of Scar-DUF grown in M9 medium.

**Movie S1. Molecular dynamics analysis indicate that the $BR_{185-387}$-DNA complex is kept through non-specific interactions over a simulation period of 50 ns**

Starting from the docked conformation, the complex appeared to be flexible but stable with the RMSD of the docking interface (atoms N, CA, C for the protein and P for DNA) in the range of 0.3 nm from the starting conformation. This is comparable to the RMSD of protein backbone atoms alone. The relatively large RMSD is mainly due to the relative movements of the monomers with respect to each other, whereas global backbone RMSD, including all backbone atoms, for individual monomers stays below 0.2 nm. Although the DNA structure tends to be distorted by the protein, many protein-DNA contacts are well preserved throughout the simulation. Most interactions (over 80%) are non-specific involving DNA backbone and sugars. Many such interactions, in particular the ones involving the phosphates are preserved throughout simulations (>90% of snapshots), whereas contacts involving bases are more labile. There is no clear indication of protein sliding along DNA, although this can be expected from the non-specificity of most contacts.

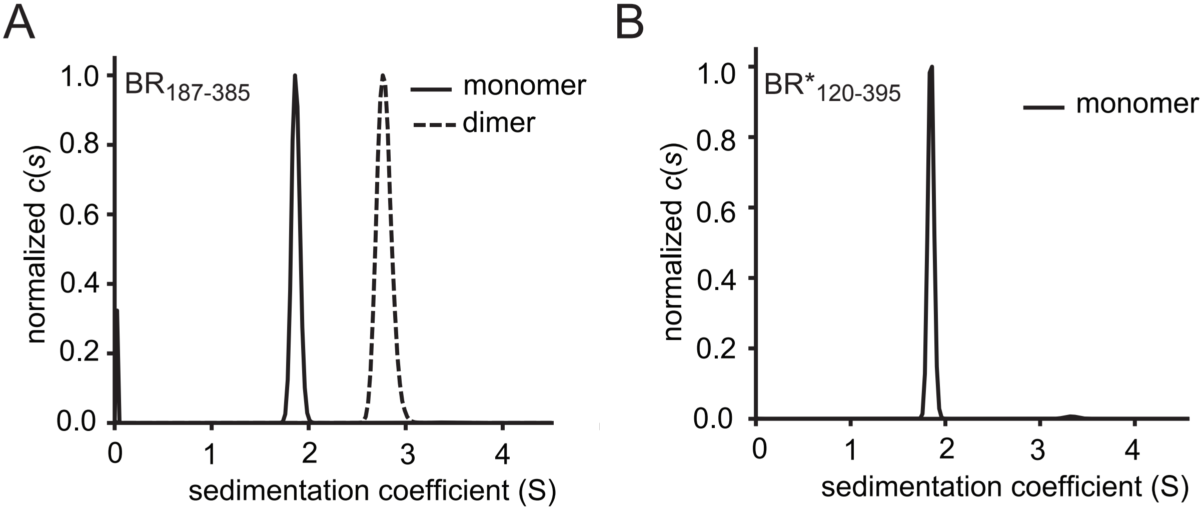
A
BR187-385
monomer
dimer
normalized c(s)
1.0
0.8
0.6
0.4
0.2
0.0
0
1
2
3
4
sedimentation coefficient (S)
B
BR*120-395
monomer
normalized c(s)
1.0
0.8
0.6
0.4
0.2
0.0
0
1
2
3
4
sedimentation coefficient (S)

FIGURE S2

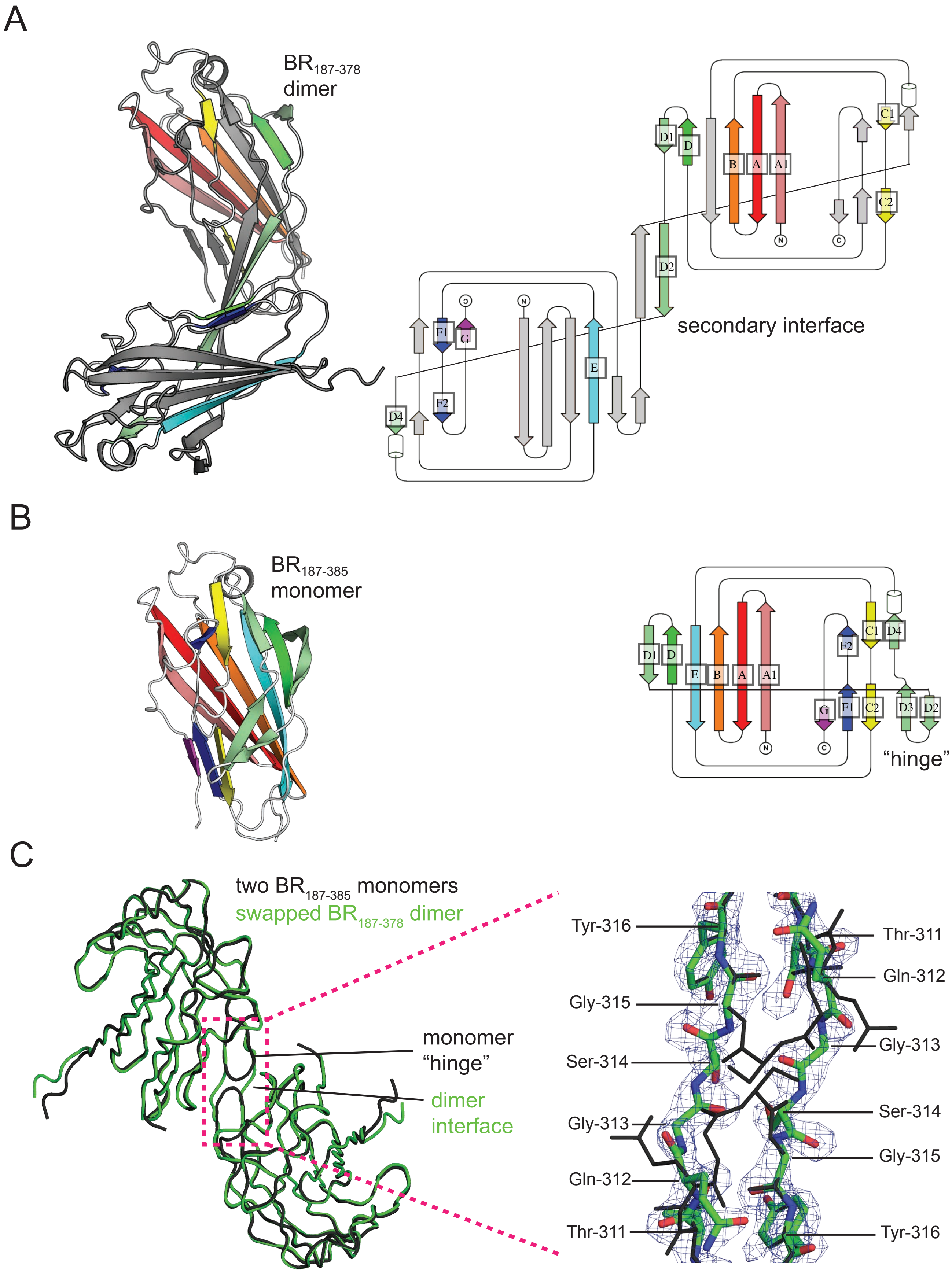
A
BR187-378
dimer
secondary interface
B
BR187-385
monomer
“hinge”
C
two BR187-385 monomers
swapped BR187-378 dimer
monomer
“hinge”
dimer
interface
Tyr-316
Gly-315
Ser-314
Gly-313
Gln-312
Thr-311
Thr-311
Gln-312
Gly-313
Ser-314
Gly-315
Tyr-316

FIGURE S3

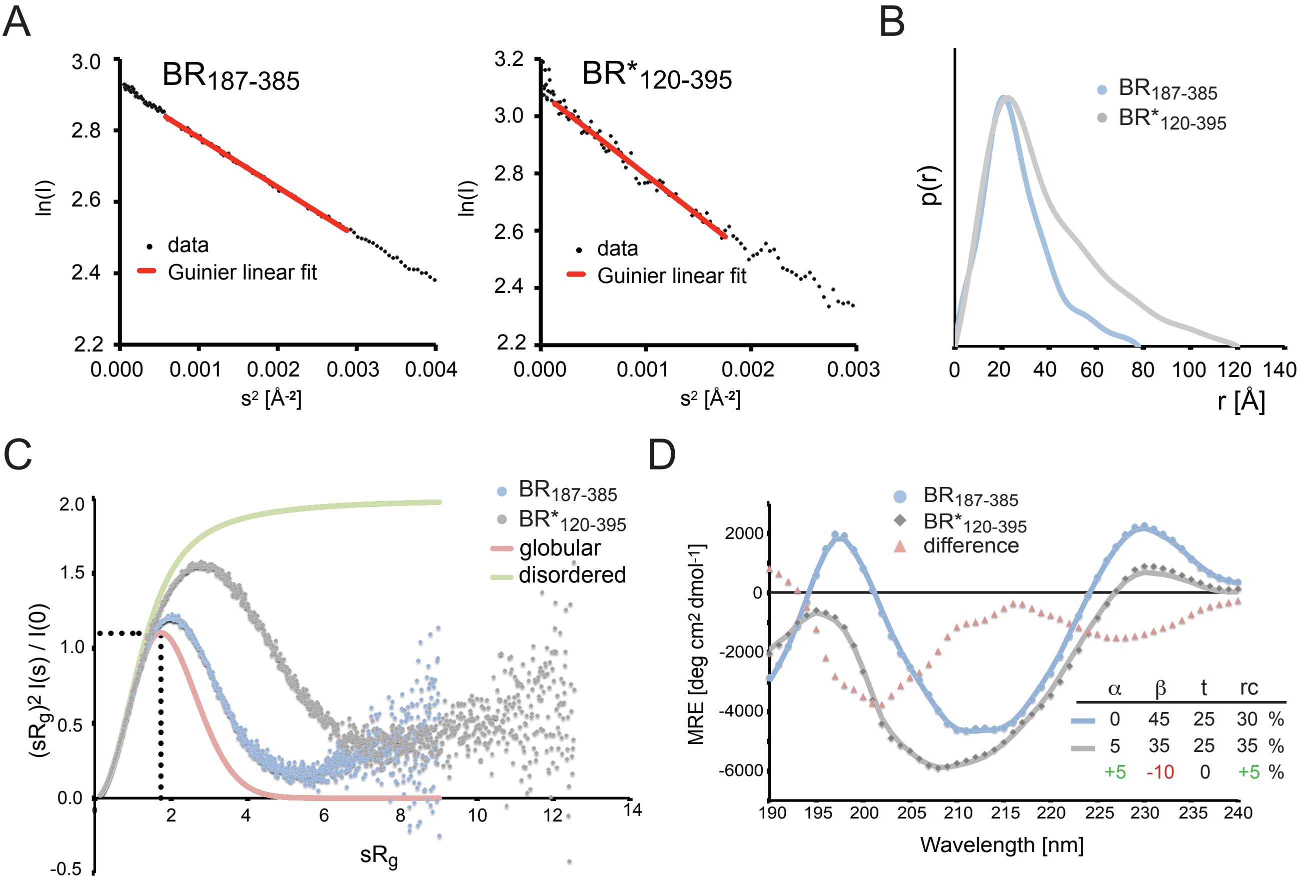
A
BR187-385
ln(I)
data
Guinier linear fit
s² [Å⁻²]
BR*120-395
B
p(r)
r [Å]
C
(sRg)² I(s) / I(0)
sRg
globular
disordered
D
MRE [deg cm² dmol⁻¹]
Wavelength [nm]
difference
α β t rc
0 45 25 30 %
5 35 25 35 %
+5 -10 0 +5 %

FIGURE S4

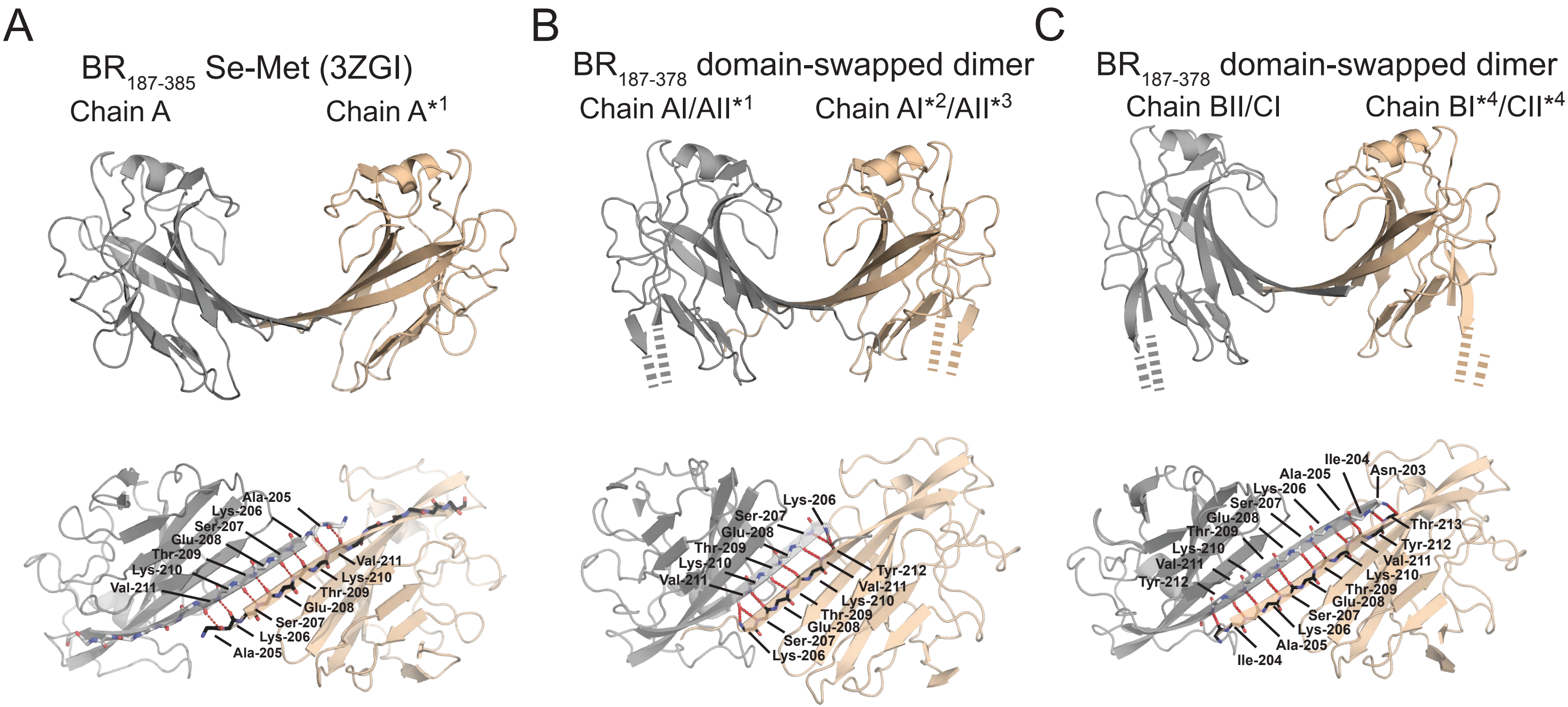
A
BR187-385 Se-Met (3ZGI)
Chain A
Chain A*1
B
BR187-378 domain-swapped dimer
Chain AI/AII*1
Chain AI*2/AII*3
C
BR187-378 domain-swapped dimer
Chain BII/CI
Chain BI*4/CII*4
Ala-205
Lys-206
Ser-207
Glu-208
Thr-209
Lys-210
Val-211
Val-211
Lys-210
Thr-209
Glu-208
Ser-207
Lys-206
Ala-205
Lys-206
Ser-207
Glu-208
Thr-209
Lys-210
Val-211
Tyr-212
Val-211
Lys-210
Thr-209
Glu-208
Ser-207
Lys-206
Ile-204
Asn-203
Ala-205
Lys-206
Ser-207
Glu-208
Thr-209
Lys-210
Val-211
Tyr-212
Thr-213
Tyr-212
Val-211
Lys-210
Thr-209
Glu-208
Ser-207
Lys-206
Ala-205
Ile-204

FIGURE S5

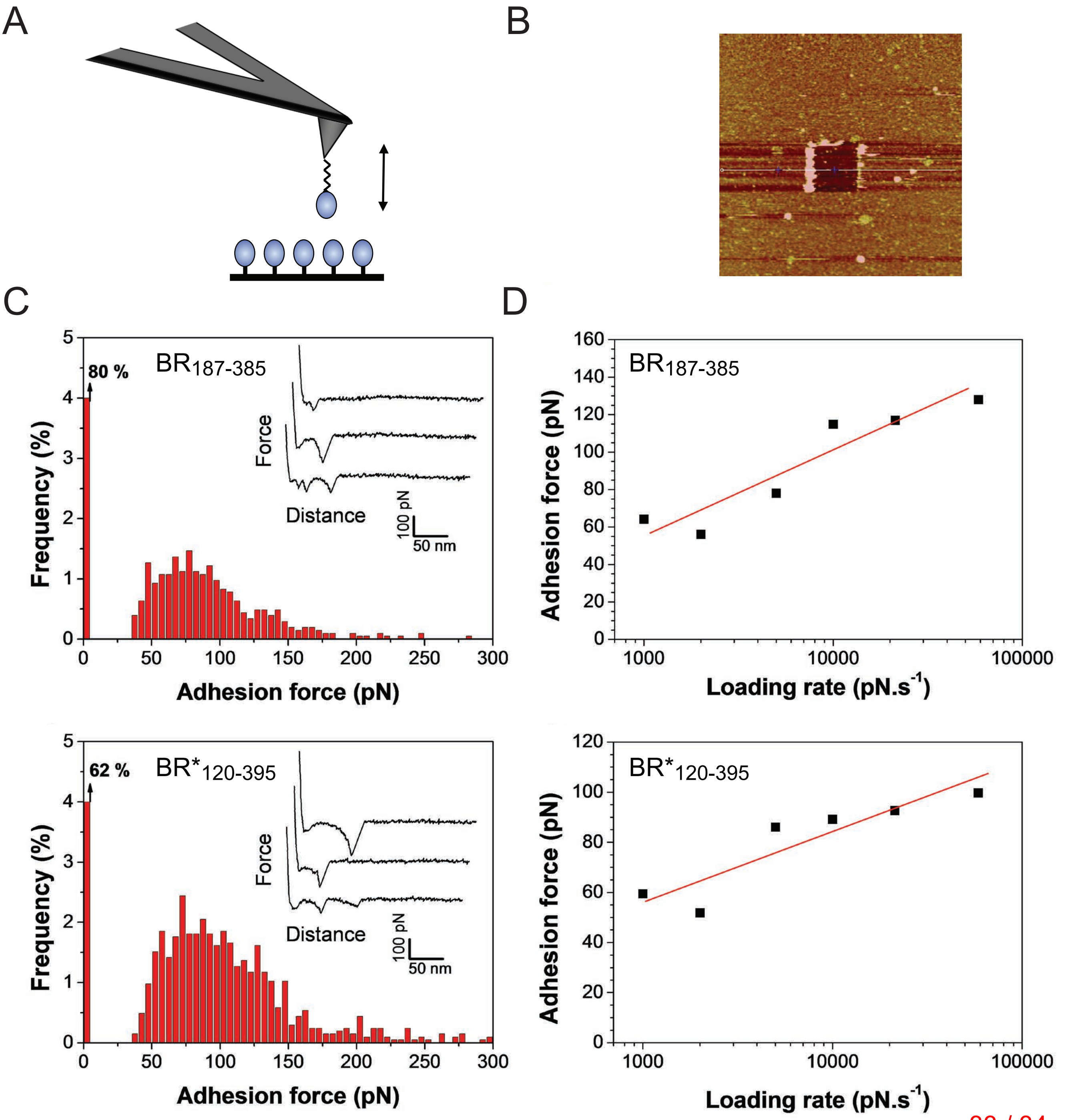
A
B
C
D
80 %
BR187-385
Force
Distance
100 pN
50 nm
Frequency (%)
Adhesion force (pN)
62 %
BR*120-395
Adhesion force (pN)
Loading rate (pN.s-1)
0
50
100
150
200
250
300
1000
10000
100000

FIGURE S6

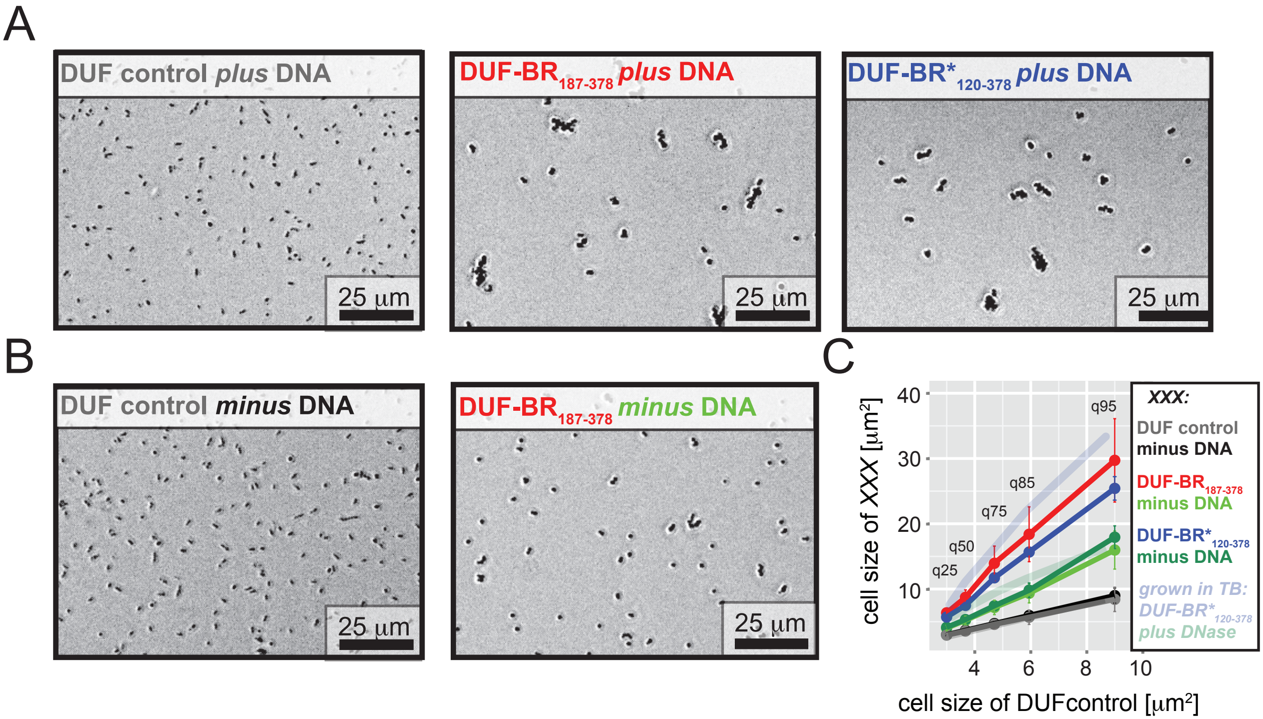
A
DUF control plus DNA
DUF-BR187-378 plus DNA
DUF-BR*120-378 plus DNA
25 μm
B
DUF control minus DNA
DUF-BR187-378 minus DNA
25 μm
C
cell size of XXX [μm²]
cell size of DUFcontrol [μm²]
q25
q50
q75
q85
q95
XXX:
DUF control minus DNA
DUF-BR187-378 minus DNA
DUF-BR*120-378 minus DNA
grown in TB: DUF-BR*120-378 plus DNase
